# Discovery of novel L-type voltage-gated calcium channel blockers and application for the prevention of inflammation and angiogenesis

**DOI:** 10.1186/s12974-020-01801-9

**Published:** 2020-04-25

**Authors:** Madhu Sudhana Saddala, Anton Lennikov, Anthony Mukwaya, Yan Yang, Michael A. Hill, Neil Lagali, Hu Huang

**Affiliations:** 1 Department of Ophthalmology, School of Medicine, University of Missouri-Columbia, 1 Hospital Drive, MA102C, Columbia, MO 65212 USA; 2 Department of Ophthalmology, Institute for Clinical and Experimental Medicine, Faculty of Health Sciences, Linköping University, Linköping, Sweden; 3 Dalton Cardiovascular Research Center, University of Missouri-Columbia, Columbia, MO USA

**Keywords:** Angiogenesis, Calcium, Inflammation, L-VGCC, Microglia, Neovascularization, Pharmacophore, Retina, Smooth muscle cells

## Abstract

**Background:**

The ways in which microglia activate and promote neovascularization (NV) are not fully understood. Recent in vivo evidence supports the theory that calcium is required for the transition of microglia from a surveillance state to an active one. The objectives of this study were to discover novel L-type voltage-gated channel (L-VGCC) blockers and investigate their application for the prevention of inflammation and angiogenesis.

**Methods:**

Pharmacophore-based computational modeling methods were used to screen for novel calcium channel blockers (CCBs) from the ZINC compound library. The effects of CCBs on calcium blockade, microglial pro-inflammatory activation, and cell toxicity were validated in BV-2 microglial cell and freshly isolated smooth muscle cell (SMC) cultures. Laser-induced choroidal neovascularization (NV) and the suture-induced inflammatory corneal NV models of angiogenesis were used for in vivo validation of the novel CCBs. CX3CR1^gfp/+^ mice were used to examine the infiltration of GFP-labeled microglial cells.

**Results:**

We identified three compounds from the ZINC database (Zinc20267861, Zinc18204217, and Zinc33254827) as new blockers of L-type voltage-gated calcium channels (L-VGCC) using a structure-based pharmacophore approach. The effects of the three CCBs on Ca^2+^ influx into cells were verified in BV-2 microglial cells using Fura-2 fluorescent dye and in freshly isolated SMCs using the voltage-patch clamp. All three CCBs reduced microglial cell migration, activation stimulated by lipopolysaccharide (LPS), and reduced the expression of the inflammatory markers NF-κB (phospho-IκBα) and cyclooxygenase-2 (COX-2) as well as reactive oxygen species. Of the three compounds, we further examined the in vivo activity of Zinc20267861. Topical treatment with Zinc20267861 in a rat model of suture-induced inflammatory cornea neovascularization demonstrated efficacy of the compound in reducing monocyte infiltration and overall corneal NV response. Subconjunctival administration of the compound in the choroidal NV mouse model effectively prevented CNV and microglial infiltration.

**Conclusions:**

Our findings suggest that the novel CCBs identified here are effective anti-inflammatory agents that can be further evaluated for treating NV disorders and can be potentially applied in the treatment of ocular inflammatory and pathological angiogenetic disorders.

## Introduction

Microglia (MG), which are the resident immune cells in the central nervous system (CNS), play a vital role in maintaining homeostasis of the CNS by mediating several effects, such as synaptic pruning, tissue repair, and host defense. However, MG activation and dysregulation partially mediated by calcium (Ca^2+^) channel activity are involved in the pathogenesis of stroke, CNS infections, and neurodegenerative diseases, including Alzheimer’s disease (AD) [1], Parkinson’s disease (PD) [2, 3], multiple sclerosis (MS) [4, 5], and macular degeneration (MD) [6]. The transition of microglia from a surveillance to an active state requires a significant phenotypic/functional change that involves Ca^2+^ ion signaling, as indicated in recent in vivo studies [7].

Spontaneous Ca^2+^ transients are rarely detectable in microglia under physiological conditions but are substantially upregulated in pathological conditions as well as in cell culture. Many ionotropic and metabotropic receptors in the plasma membrane regulate the intracellular free Ca^2+^ concentration through entry into the cytosol from the extracellular environment and/or via store-operated Ca^2+^ entry (SOCE). Therefore, intracellular calcium signaling serves as the common pathway required for the functioning of activated microglia, including their proinflammatory signals, which leads to the expression of the following cytokines and neurotoxic factors: tumor necrosis factor-alpha (TNF-α), cyclooxygenase (COX-2), interleukin-1 beta (IL-1β), and reactive oxygen species (ROS). Microglia are known to express L-type voltage-gated Ca^2+^ channels (L-VGCC), which are upregulated under pathological conditions [8]. The L-VGCC is a multisubunit complex of heteromeric proteins consisting of the pore-forming alpha-1 (α1) subunit, disulfide-linked transmembrane complex of alpha-2 (α2), intracellular beta (β) subunit, delta (Δ), and gamma (γ) subunits [9]. The pore-forming α1 subunit directs the channel activity, which displays significant pharmacological and electrophysiological properties. The pore-forming α1 subunit of L-VGCC folds from a single polypeptide chain self-possessed of four repeats (I–IV); every repeat is created by six transmembrane segments (S1–S6). The S1–S4 segments have a voltage-sensing domain, an outer helix S5, an inner helix S6, and a membrane dividing P-loop linking S5–S6. The EEEE ring (selecting filter glutamates) in four repeats (I–IV) of L-VGCC in P-loops forms the selectivity filter for Ca^2+^ ions [10].

Modulation of microglial via L-VGCC has recently shown great promise as a therapeutic strategy in certain conditions [11, 12]. In the current work, we used the amlodipine structure as a model to conduct pharmacophore-based virtual screening and a docking simulation to identify compounds with higher binding energy to L-VGCC, as well as those with absorption, distribution, metabolism, and excretion (ADME) indexes greater than the reference substance amlodipine, which is a known calcium channel blocker, to create a third generation of L-VGCC blocking agents. We screened the known substance databases of modeling and simulation experiments. In vitro, we used a mouse immortalized microglial cell line (BV-2) and freshly isolated SMC cultures, both of which are known to naturally express L-VGCC, to evaluate the safety and efficacy of the identified substances. We further evaluated the capacity of the substance to alter the electric potential of BV-2 cells. The substance with the highest binding energy to L-VGCC was evaluated in vivo using two clinically relevant animal models: mouse laser-induced neovascularization (CNV) [13] and suture-induced inflammatory corneal neovascularization (SI-CNV) in rats [14–16]. We used these models to evaluate the therapeutic potential of the designed compounds to inhibit pathological angiogenesis by targeting the activation and migration of inflammatory cells.

## Methods

### In silico protein model preparation

The L-VGCC model protein was taken from our previous work [10]. Briefly, construction of the transmembrane region of the model was achieved by using MODELLER v9.13 (https://www.salilab.org). The structural model of the human LCC was built using the recently reported 3.20 Å crystal structure of KcsA6 (Protein Data Bank (PDB) entry code 1BL8) as a structural template. The sequence of the human LCC pore region alpha-1c subunit (Cav1.2, CAC1C_HUMAN) was retrieved and aligned with data from the SWISS-PROT database. Amino acid sequences of S5, S6, and the P-loops in between the four repeats (I–IV) (271–405, 654–753, 1052–1185, and 1411–1524, respectively) were used for sequence alignment with the amino acid sequence of KcsA. The sequence alignment file was used as input for the MODELLER v9.13 program to model the protein, and molecular dynamics was performed using the Gromacs tool (https://www.gromacs.org). The stereochemical quality and structural integrity of the model were tested with the RAMPAGE (http://mordred.bioc.cam.ac.uk/~rapper/rampage.php), ERRAT (https://servicesn.mbi.ucla.edu/ERRAT/), ProSA (https://prosa.services.came.sbg.ac.at/prosa.php), and Verify3D (https://servicesn.mbi.ucla.edu/Verify3D/) tools [10, 17].

### Binding site analysis

The modeled L-VGCC was used for prediction of the active site (drug binding site) using the CASTp (Computed Atlas of Surface Topography of Proteins) server (http://sts.bioe.uic.edu/castp/). It locates all likely binding pockets, and the algorithm determines the binding pocket and possible cavities in a solvent-accessible surface area. We uploaded the L-VGCC as input to predict the ligand binding sites. The CASTp server predicted the key amino acids for binding interactions to the inhibitors.

### Structure-based pharmacophore model design

Amlodipine was docked to the active site region of the L-VGCC model and the protein-ligand complex used for pharmacophore design. The structure-based pharmacophore model design for the L-VGCC model and amlodipine complex was generated by the LigandScout (LS) software 4.1 (http://www.inteligand.com/ligandscout/). LigandScout is a computer software that allows the creation of three-dimensional (3D) pharmacophore models from structural data of macromolecule-ligand complexes. It incorporates a complete definition of 3D chemical features (such as hydrogen bond donors, acceptors, lipophilic areas, positively and negatively ionizable chemical groups) that describe the interaction of a bound small molecule (ligand) and the surrounding binding site of the macromolecule [18]. Alternative hydrogen bond donor and acceptor sites are considered simultaneously on the protein within the limits of geometric constraints. Exclusion volume spheres were added to the structure-based models onto coordinates defined by protein side-chain atoms to characterize inaccessible areas for any potential ligand [19]. In this current work, the L-VGCC modeled protein complex with a channel blocker amlodipine was employed as the initial model to design a structure-based pharmacophore model finding the pharmacophoric features in the active site. These features were then clustered, and the most representative features were selected and included in the pharmacophore model for virtual screening in the Zinc database.

### Pharmacophore-based virtual screening

Virtual screening of chemical databases can enable the identification of novel leads that are suitable for further development. The database searching methodology provides the advantage that the retrieved compounds can be obtained easily for biological testing when compared to any de novo design [20]. A molecule must fit to all the features of the pharmacophore model that are used as a 3D query in a database search to be retained as a hit. In our study, we performed a search of the Zinc database, which allocates the client to download molecules, structures from a diversity of dealers as SDF files based on the structure-based similarity. The screened compounds were uploaded into the LigandScout software 4.1 (http://www.inteligand.com/ligandscout/), and the 3D structure of all the compounds was built with the MMFF94x force field to create a local database for screening. The local database was assigned to the pharmacophore model and screened. Based on the pharmacophore feature, 14 compounds (hits) met the pharmacophore model. These 14 hits were used for docking simulation analysis.

### Docking simulation

Molecular docking is a computational procedure that attempts to predict the noncovalent binding of protein and ligand efficiently. Docking programs use a scoring function to approximate the standard chemical potential of the system. All calculations were performed with the docking program AutoDock Vina [21]. The choice of AutoDock Vina as docking software was directed by its ability to find bioactive conformations with an excellent level of accuracy. The AutoDock graphical interface AutoDock Tools 46, 47 was used to keep polar hydrogens and add partial charges to the proteins using Kollman United charges. The search space was included in a box of 24 × 24 × 24 Å, centered on the binding site of the ligands and nicotinamide cofactor. Pharmacophoric hits were docked with the refined LCC model active site. The Lamarckian genetic algorithm was utilized as the max number of energy evaluation (25,000,000), max number of generations (27,000) [22], number of individual populations (150), crossover rate (0.8), Cauchy beta (1.0), Gene mutation rate (0.02), and GA window size (11.0). The grid was placed to cover the whole protein due to the multibinding pocket (i.e., pore region) at *X* = 4.67, *Y* = − 57.43, *Z* = 99.52, and dimension Å at *X* = 88.72, *Y* = 97.57, *Z* = 99.52, and exhaustiveness 8. The results were clustered in accordance with their root mean square deviation (RMSD) values and binding energies, which were considered by the AutoDock scoring function. The PyMol molecular viewer tool (http://www.pymol.org/) was employed to explore the docked structures.

### Analysis of pharmacokinetic profiles

The best compounds were further analyzed for their various molecular properties and to predict the bioactivity score using the Molinspiration software version v2016.03 (www.molinspiration.com). Molecular properties such as the partition coefficient (LogP), topological polar surface area (TPSA), hydrogen bond donors (n-OHNH) and acceptors (n-ON), rotatable bonds (n-rotb), number of atoms, molecular weight (MW), and violations of Lipinski’s rule of five were calculated to evaluate the drug-likeness of the synthesized compounds [23]. The method for calculation of clogP was developed by Molinspiration (miLogP2.2e-2005) based on group contributions and correction factors by fitting the calculated log P to the experimental log P for a training set of, more than twelve thousand, drug-like molecules. The molecular polar surface area (PSA) was calculated based on the methodology published by Ertl et al. as a sum of fragment contributions [24]. The maps of molecular lipophilicity potential (MLP) and polar surface area (PSA) were viewed in the Molinspiration Galaxy 3D Structure Generator (ver. 2010.02 beta) using an optimized structure generated by the semi-empirical molecular orbital PM3 method [24] which also predicts various bioactivity scores for drug targets, including enzymes and nuclear receptors, kinase inhibitors, G protein-coupled receptor (GPCR) ligands, and ion channel modulators. The drug-likeness properties and bioactivity scores of the best compounds were compared with those of the standard drugs indomethacin and aspirin. The absorption percentage (% Ab) was also calculated by the method described by Zhao et al. using the succeeding formula %Ab = 109 − [0.345 × TPSA] [25].

### ADMET profiles

The ADMET (absorption, distribution, metabolism, elimination, and toxicity) profiles were predicted for the five best compounds using the admetSAR server. The ADMET structure-activity relationship server (admetSAR) is a comprehensive knowledge tool for predicting ADMET properties of drug candidates and environmental chemicals (http://www.admetexp.org). We calculated the physicochemical properties and the ADMET-associated profiles. The physicochemical properties include MW, Log P, number of hydrogen bond acceptors and donors, and TopoPSA. There are more than 45 kinds of ADMET-associated properties, proteins, species, and organisms, such as water solubility, human intestinal absorption, oral bioavailability, blood-brain barrier penetration, P-glycoprotein substrate and inhibitor, renal organic cation transporter, plasma protein binding, volume of distribution, CYP450 substrates and inhibition (CYP1A2, 2C9, 2C19, 2D6, and 3A4), drug-induced liver injury, human Ether-a-go-go-Related gene (hERG) inhibition, rat acute toxicity, skin sensitivity, AMES mutagenicity, carcinogens, fish toxicity, Tetrahymena pyriformis toxicity, honey bee toxicity, quail toxicity, reproductive toxicity, biodegradability, bioconcentration factors, and others. We calculated the detailed biological endpoints, such as Ki, IC_50_ (half maximal inhibitory concentration), LC_50_ (median lethal concentration), LD_50_ (median lethal dose), IGC_50_ (50% growth inhibitory concentration), AC_50_ (the compound concentration leads to 50% of the activity of an inhibition control), EC_50_ (half-maximal effective concentration), and TD_50_ (median toxic dose), respectively, for the five best compounds including the reference compound (amlodipine).

### Compounds and preparation of experimental solutions

Compounds Zinc20267861 (MolPort-000-490-667; Mwt 485.364 g/l; MolPort), Zinc18204217 (R631116; Mwt 502.745 g/l; DiscoveryCPR), and Zinc33254827 (MolPort-010-738-759; Mwt 395.459 g/l; MolPort) with the best in silico properties and predicted binding to L-VGCC were purchased from MolPort and DiscoveryCPR and further validated for in vitro toxicity and Ca^2+^ blocking activity estimation. L-VGCC blocker Amlodipine (AMLD; Sigma; Mwt 567.1 g/l) and L-VGCC agonist BayK8644 (B112-5MG, Sigma Mwt 356.3 g/l) were used as a reference substances. Substances were dissolved in a 100% dimethyl sulfoxide (DMSO) 10 mM concentration stock solution, and a working solution of 1 mM was prepared by dissolving 1 part of the stock solution in 9 parts of phosphate-buffered saline (PBS), resulting in 10% DMSO in PBS solvent. Then, working solution was mixed with cell culture medium to produce the experimental concentrations: Zinc20267861 (2.0 μM), Zinc18204217 (2.0 μM), Zinc33254827 (2.5 μM), and AMLD (3.5 μM). For patch-clamp experiments are as follows: Zinc20267861 (0.5–5.0 μM), amlodipine (2 μM), and Ca^2+^ agonist BayK8644 (B112-5MG, Sigma; 1 μM). As a control, 10% DMSO in PBS solvent was used by dissolving 1 part of pure DMSO in 9 parts of PBS and using 1 μl per 1 ml of media. Lipopolysaccharide (LPS) (Sigma) 1 μg/ml and interleukin 4 (IL-4) (PMC0045; Thermo Fisher) 20 ng/ml were used to stimulate BV-2 cells.

### Cells and cell culture conditions

The immortalized mouse microglial cell line BV-2 (EOC-20; CRL-2469; Lot: 70005904; ATCC), which is derived from the C3H/HeJ female mouse, was used at passage 7. BV-2 cells were cultured in Dulbecco’s Modified Eagle Medium (DMEM) (Gibco, Thermo Fisher Scientific) supplemented with 10% fetal bovine serum (FBS; Gibco, Thermo Fisher Scientific), 1% Pen/Strep (Gibco, Thermo Fisher Scientific), and 20% LADMAC conditioned medium (LCM). Mouse bone marrow derived macrophage cell line LADMAC (CRL-2420; Lot: 63407846; ATCC) was used as a source of crude CSF-1 to supplement BV-2 cell growth. LADMAC cells were grown to confluency in complete DMEM; then, the media was replaced with fresh complete DMEM, and 24 h later, conditioned medium was collected and centrifuged at 5000×*g* for 10 min to remove cells and debris, resulting in supernatants that were further filtered through 0.2-μm-pore-size and syringe-driven Target2™ filter (F2513-2, Thermo Fisher Scientific). The resulting LCM was stored at – 20 °C until use. HEK293FT (R70007, Invitrogen, Thermo Fisher Scientific, MA, USA) cells at passage 20 were cultured in complete DMEM. In the experiments involving stimulation of BV-2 cells with lipopolysaccharide (LPS) or IL-4, BV-2 cells were grown for 12 h in FBS-free medium (DMEM, 1% Pen/Strep).

### Fluorescent and confocal microscopy

Fluorescent images were acquired with a Leica SP8 laser confocal microscope (Leica AG, Wetzlar, Germany) and LSM 700 laser confocal microscope (Carl Zeiss, Oberkochen, Germany). Calcein AM-stained BV-2 cell migration data and ROS signaling were acquired using the EVOS FL imaging system (Thermo Fisher Scientific).

### BV-2 cell death assay

BV-2 cells at a density 3 × 10^5^ were seeded into a 24-well plate. Following cell attachment, cultures were treated with accending concentrations of Zinc20267861, Zinc18204217, and Zinc33254827 (2, 5, 10 μM) for 24 h. For the negative control, cells were treated with 1 μl/ml of 10% DMSO in PBS, and for the positive control, cells were treated with 0.05% Triton-X100 for 30 min, a sufficient time to induce membrane damage and cell death, but short enough to preserve cell morphological integrity for staining. Then, active components of the ReadyProbes Cell Viability Imaging Kit (Blue/Green) (R37609, Invitrogen, Thermo Fisher Scientific) at 20 μl/ml were added to the culture medium and incubated for 30 min.

### Fura-2 calcium signal detection

BV-2 or HEK293FT cells were seeded into a 24-well plate at a density of 3 × 10^5^, and following. Following attachment, cells were treated with Zinc20267861, Zinc18204217, and Zinc33254827 or amlodipine for 10 min. Then, Fura-2-acetoxymethyl ester (Fura-2AM; F1201, Invitrogen, Thermo Fisher Scientific) at 1 μg/ml was added to the culture medium, and imaging was performed with the LSM 700 inverted laser confocal microscope with a 380 nm excitation and 480–500 nm emission filter, corresponding to Fura-2 calcium-free excitation/emission peaks. The presence of a signal indicated the presence of unconjugated Fura-2 in the cells, revealing low levels of Ca^2+^ in the cytoplasm. Fura-2 saturation with Ca^2+^ resulted in an excitation shift to 340 nm and loss of the signal at 380 nm, indicating the presence of Ca^2+^. The negative control consisted of untreated BV-2 cells stained with Fura-2, while the positive control consisted of known L-VGCC amlodipine. Assay specificity control has utilized the lack of L-gated calcium channels in HEK293FT cells, which thus are expected not to respond to amlodipine L-VGCC blocking effect and to fail to produce a signal in the 380 nm excitation, 480–500 nm emission range.

### Electric cell-substrate impedance sensing

BV-2 cells were seeded on 8-well electric cell-substrate impedance sensing (ECIS) arrays and grown to confluence in LCM medium. An ECIS Zθ instrument (Applied Biophysics, USA) was used to measure electrical resistance changes (at 4 kHz) and impedance (at 16 kHz) in confluent BV-2 cell cultures treated with 10 μM of Zinc20267861, Zinc18204217, and Zinc33254827 or amlodipine. The changes in BV-2 resistance and impedance were monitored in real-time every 120 s and recorded on the ECIS software for MAC OSX (Applied Biophysics, Troy, NY).

### Isolation of mesenteric small arteries, preparation of primary single smooth muscle cells, and whole-cell Ca^2+^ channel recordings

Single smooth muscle cells (SMCs) from second and third order arterioles (2A/3A) were isolated as previously described [26]. In brief, mouse small intestine was excised and pinned flat for dissection of small mesenteric arteries at 4 °C HBSS solution. Dissected vessel segments were transferred to a 1-ml tube containing low-Ca^2+^ physiological salt solution (PSS; in mM): NaCl, 144; KCl, 5.6; CaCl2, 0.1; MgCl2, 1.0; Na2HPO4, 0.42; HEPES, 10; sodium pyruvate, 2; and 1 mg/ml BSA at room temperature (RT) for 10 min. The solution was decanted and replaced with a similar solution containing 26 U/ml papain and 1 mg/ml dithiothreitol (DTT). The vessels were incubated for 30 min at 37 °C with occasional agitation and then transferred to a new tube containing low-Ca^2+^ PSS, 1.95 U/ml collagenase (Type H FALGPA), 1 mg/ml soybean trypsin inhibitor, and 75 U/ml elastase, and incubated for 5 min at 37 °C . After further digestion, the remaining fragments were gently rinsed (2–3×) with low-Ca^2+^ PSS and gently triturated using a fire-polished Pasteur pipette to release single cells. Spindle-shaped SMCs were used for electrophysiological patch-clamp studies within approximately 4 h of isolation. SMCs were seeded as previously described [27, 28]. SMC L-VGCC currents were recorded using a standard whole-cell patch-clamp technique [26, 27]. Briefly, cells were superfused with physiological saline solution (PSS) containing tetraethylammonium chloride (TEA-Cl), 138 mM; CaCl2, 0.1 mM; MgCl2, 1 mM; HEPES, 10 mM; glucose, 5 mM; and barium chloride (BaCl2), 20 mM pH 7.35 (Osm ~ 300 Osm/L). The pipette solution consisted of the following: CsCl, 120 mM; TEA-Cl 10 mM; EGTA, 10 mM; MgCl2, 1 mM; HEPES, 15 mM; Na2-ATP, 5 mM; Tris-GTP, 0.5 mM; and CaCl2, 0.1 mM pH 7.2. L-VGCC response curves were obtained by using a holding potential of − 70 mV, with step changes in potential from − 60 to + 60 mV and a duration of 400 ms. Ca^2+^ currents are presented as picoamperes (pAs).

### Immunohistochemistry

BV-2 cells were seeded at a density of 5 × 10^3^ cells per well on a Millicell EZ slide (Millipore, Billerica, MA, USA) and grown to 80% confluence in LCM. After 12 h of serum starvation in FBS-free DMEM, cells were treated with Zinc20267861, Zinc18204217, and Zinc33254827 and incubated for 12 h. Following incubation, BV-2 cells were stimulated with 1 μg/ml LPS (Sigma) for 24 h. Samples were fixed with 2% formaldehyde (Sigma-Aldrich) for 10 min, permeabilized with 0.2% Triton-X100 (Sigma) in PBS for 15 min, and then blocked with 5% NGS (Thermo Fisher Scientific) for 1 h at RT. The samples were incubated with COX-2 (MA5-14568; 1:1000; Thermo Fisher Scientific) and p-IκB-α (B-9) (sc-8404; 1:100; Santa Cruz Biotechnology, CA, USA) antibody overnight; then, they were visualized DyLight 647 and 488, 1:1000 (Thermo Fisher Scientific). Sections were counterstained with 4′,6-diamidino-2-phenylindole (DAPI) 1:5000 (Sigma-Aldrich) and mounted ProLong Diamond antifade reagent (Invitrogen, Thermo Fisher Scientific).

### Actin-F staining

BV-2 cells were grown to 80% confluence as described above and incubated with Zinc20267861, Zinc18204217, and Zinc33254827 at 1 μg/ml for 12 h. Samples were fixed with 2% formaldehyde (Sigma-Aldrich) for 10 min, permeabilized with 0.2% Triton-X100 (Sigma) in PBS for 15 min, and then blocked with 5% NGS (Thermo Fisher Scientific) for 1 h at RT. Then, the samples were incubated with phalloidin red (1:100; Thermo Fischer Scientific) for 1 h at RT and washed with PBS. Following washing, samples were counterstained with DAPI 1:5000 (Sigma-Aldrich) and mounted with ProLong Diamond antifade reagent (Thermo Fisher Scientific).

### BV-2 migration assay

BV-2 cells were grown to confluence in a 6-well-plate with complete medium supplemented with 20% LCM. A scratch along the diameter of the well was introduced using a sterile razor, the detached cells were then removed by washing with PBS, supplied with fresh complete culture medium (20% LCM), and treated with 1 μg/ml of Zinc20267861, Zinc18204217, and Zinc33254827. DMSO-treated wells and 1 μg/ml amlodipine-treated wells were used as controls. Ninety-six hours after treatment, the cells were stained with Calcein AM (C3100MP; Thermo Fisher Scientific) at 1 μg/ml and imaged using an EVOS fluorescence microscope (Thermo Fisher Scientific) with an excitation wavelength of 488 nm. The starting point was determined based on the original imprint of the blade at the bottom of each well. The distance from the starting point to where the furthest cell migrated was measured and quantified. Three images were obtained from each well, and the results were averaged.

### RNA isolation and quantitative real-time PCR (qRT-PCR)

BV-2 cells stimulated with LPS or IL-4 were washed with PBS, and total RNA was extracted using the RNeasy Plus Mini Kit (Qiagen) according to the kit protocol. The extraction kit lysis buffer was supplemented with 20 μl of 2 M dithiothreitol (DTT). RNA was analyzed for quality and quantified using a NanoDrop One (Thermo Fisher Scientific) and reverse-transcribed to cDNA using Maxima™ H Minus cDNA Synthesis Master Mix with dsDNase (M1682; Thermo Fisher Scientific), according to the manufacturer’s protocol (SimpliAmp Thermal Cycler, (Life Technology, MA, USA). Gene expression analysis was performed using Power SYBR Green Master Mix (Thermo Fisher Scientific) with the following mouse-specific primers: Arg-1 (forward: GGAATCTGCATGGGCAACCTGTGT, reverse: GGAATCTGCATGGGCAACCTGT-GT, reverse: AGGGTCTACGTCTCGCAAGCCA), COX-2 (forward: GCGAGCTAAGAGCTTCAGGA, reverse: CAGACGCCACTGTCGCTTT), and Cyclophilin (forward: CAGACGCCACTGTCGCTTT, reverse: TGTCTTTGGAACTTTGTCTG) on a Quant Studio 3 RT-PCR system (Applied Biosystems, CA, USA). The relative expression values of target genes were normalized to cyclophilin as the housekeeping gene, and the fold change was calculated using the relative quantification (2−ΔΔCT) method. Four biological replicates per treatment group were run with three technical replicates.

### ROS signaling

Dihydroethidium (DHE, Sigma-Aldrich, St. Louis, MO, USA), an oxidative red fluorescent dye, was used for cytosolic superoxide anion (O^2−^) detection by oxidation [29]. BV-2 cells were seeded into the 24-well plate in LCM and grown to 80% confluence and, following 12 h of growth in DMEM, the cells were treated with 1 μg/ml of Zinc20267861, Zinc18204217, and Zinc33254827 and stimulated with LPS 1 μg/ml for 24 h. Then, the medium was replaced with fresh DMEM, and DHE 1 μg/ml was added to the wells, followed by a 15-min incubation at 37 °C, 37 °C, 5% CO2. Then, 24-well plate was mounted on the EVOS FL imaging system (Thermo Fisher Scientific), and living cells were observed and imaged at excitation wavelengths of 350 nm (Dihydroethidium) and 595 nm (oxidized-ethidium).

### Mouse housing and breeding

Mice were used in accordance with the approved protocols by the Institutional Animal Care and Use Committee (IACUC # 9520) of the University of Missouri and the guidelines of the Association for Research in Vision and Ophthalmology (ARVO). CX3CR1^gfp/gfp^, C57BL/6J, and CX3CR1^gfp/wt^ mice (Jackson Lab, Bar Harbor, ME) were housed at the Bond Life Science Building (BLSB) of the University of Missouri. CX3CR1^gfp/wt^ mice were produced by breeding CX3CR1^gfp/gfp^ males with C57BL/6J (WT) female mice. Genomic DNA (gDNA) was extracted from tail tip material (approximately 1–2 mm in length) from C57BL/6J (C57) and CX3CR1^gfp/wt^ mice. Genotyping was performed with the assistance of Transnetyx: Outsourced PCR Genotyping Services (www.transnetyx.com) using a real-time PCR genotypic assay for the presence of the CX3CR1-GFP insert.

### Laser CNV induction and subconjunctival injection of Zinc20267861

The laser CNV model in mice was performed according to previously published procedures [30] in 6~8-week-old C57BL/6J and CX3CR1^gfp/wt^ mice. In brief, mice were anesthetized with ketamine hydrochloride (100 mg/kg body weight) and xylazine (4 mg/kg body weight), and the pupils were dilated with 1% tropicamide (Akorn, Forest Lake, IL, USA). Laser injury (75 μm spot size, 0.1-s duration, 120 mW) was performed in the 9, 12, and 3 o’clock positions of the posterior pole of the retina with the slit lamp delivery system of an Oculight GL diode laser (Iridex, Mountain View, CA, USA) and a handheld plastic cover as a contact lens. Only eyes with burns in which a bubble was produced were used in the study. The test compound was dissolved as described above and 10 μl (10 μg) injected into the eye via the subconjunctival route. Injections were given immediately after laser treatment and on a daily basis up to 7 days post-laser treatment. Equal amounts of the vehicle (10% DMSO in PBS) were injected into the control animals via the subconjunctival route.

### Fundus examination and fluorescent angiography with a retinal-imaging microscope

Mice were anesthetized intraperitoneally (i.p.) with Ketanest (ketamine; 25 mg/ml, 0.4 ml, Pfizer, NY, USA) and Dexdomitor (dexmedetomidine hydrochloride; 0.5 mg/ml, 0.2 ml, Orion Pharma, Hamburg, Germany). Pupils were dilated with 1% tropicamide (Sandoz Inc., Holzkirchen, Germany). The cornea was protected with (hypromellose ophthalmic demulcent solution) Gonak 2.5% (Akorn, Forest Lake, IL, USA) transparent gonioscopy gel. The fundus examination was performed with a Micron III retinal-imaging microscope (Phoenix Research Labs, Inc., Pleasanton, CA, USA). Following the acquisition of visible light images of the fundus, mice received a subcutaneous injection of 100 μl of 5% sodium fluorescein (Alcon Laboratories, Fort Worth, TX, USA) per animal. Fundus vascular fluorescence was observed using 488 nm excitation with a 520-nm emission filter. Fluorescent angiographies were obtained 30, 60, and 120 s after subcutaneous injection of fluorescein sodium (250 μl, 100 mg/ml, or 10% w/v). The area of fluorescence indicating leakage from the laser burns was measured and quantified by the pixel count using the Photoshop software (Adobe Inc., San Jose, CA, USA).

### Retinal pigment epithelia choroid-scleral complex (RCSC) flat mounts

Mice were euthanized by CO2 inhalation. Eyeballs were fixed with 4% paraformaldehyde (Sigma-Aldrich) for 12 h. Under an Olympus SZ-STB1 (Olympus) dissection microscope, the anterior segment tissues, vitreous, and retinas were removed to isolate the retinal pigment epithelia choroid-scleral complex (RCSC). Approximately four to eight relaxing radial incisions were created, and the remaining RCSC were incubated overnight in a blocking solution composed of 5% NGS (Thermo Fisher Scientific) with 0.01% Triton-X (Sigma-Aldrich). The RPE–choroidal-scleral complexes were then incubated with COX-2 (MA5-14568; 1:1000; Thermo Fisher Scientific) and p-IκB-α (B-9) (sc-8404; 1:100; Santa Cruz Biotechnology, CA, USA) antibody for 24 h; following washing three times for 10 min with PBS-T, the samples were incubated for 24 h with DyLight 647 and 488, 1:1000; (Thermo Fisher Scientific) and DAPI 1:5000 (Sigma-Aldrich). Following another wash with PBS-T, the whole mounts were counterstained with DAPI and mounted with ProLong Diamond antifade reagent (Thermo Fisher Scientific).

### Rat housing and suture-induced inflammatory corneal neovascularization model (SI-CNV)

Twelve to sixteen-week-old male Wistar rats (Scanbur AB, Sollentuna, Sweden) were used, with 4 animals in the treatment and control groups. The use of animals was in accordance with the ARVO Statement for the Use of Animals in Ophthalmic and Vision Research, and all procedures involving rats were approved by the Regional Animal Ethics Review Board in Linköping, Sweden (ethical permit no. 585). Animals were maintained in a licensed care facility under standard conditions (Center for Biomedical Research, University of Linköping, Sweden). Experiments were in accordance with the ARVO guidelines for the use of animals in research. The suture induced corneal neovascularisation model of inflammatory angiogenesis was performed as detailed previously [14, 31–33]. In summary, two intrastromal sutures were placed into the temporal side of the rat cornea 1.5 mm from the limbus to induce sprouting angiogenesis over 4 days.

### Topical compound preparation and treatment schedule

Zinc20267861 (5 μg/1 μl) was prepared as follows: a stock solution of 50 μg/ml was prepared in 100% DMSO. Next, one volume of DMSO was dissolved in 9 volumes of 50% PBS/distilled water solution. The resultant solution was applied topically (as eye drops) 4x/day for 4 days. Equal amounts of the vehicle (10% DMSO in 50% water/PBS) were applied to the control rats. On the fourth day, slit lamp (Micron III, Phoenix Research Laboratories) and in vivo confocal microscopy (HrT3, Heidelberg engineering, Heidelberg, Germany) data were collected and analyzed.

### Slit-lamp imaging

The rodent slit lamp (Micron III, Phoenix Research Laboratories) was used to capture images to monitor the overall neovascularization response. A semi-quantitative neovascularization score was adopted as follows: previously described [15]. Briefly, digital slit lamp images were assigned random code numbers and placed in a folder in random order. Next, a masked observer assigned a subjective score to the images based on representative slit lamp images of the scoring system, as reported by Lennikov et al. [15] (https://www.ncbi.nlm.nih.gov/pmc/articles/PMC5878206/bin/10456_2018_9594_MOESM1_ESM.docx). The vessel length from the acquired slit lamp images of the cornea was determined as reported previously [34]. Briefly, the line tool together with the area calculator (ImageJ software, National Institutes of Health, http://rsb.info.nih.gov/ij/index.html) were used to quantify the vascular area as defined by the area of the polygon with vertices demarcated by the limbus and front of neovessels, with the whole area of the polygon determined from the suture position to the limbus and considered as 100%. The vascular area was then calculated for each rat in each experimental group as a percentage of the whole area.

### In vivo confocal microscopy (IVCM)

In vivo confocal microscopy (IVCM) (Heidelberg Retinal Tomograph 3 with Rostock Corneal Module HRT3-RCM, Heidelberg Engineering, Germany) was used to monitor cellular infiltration as detailed previously [32]. Using the ImageJ cell counting tool (ImageJ software, National Institutes of Health, http://rsb.info.nih.gov/ij/index.html), the number of inflammatory cells/IVCM field was counted in at least four image sequences selected/rat.

### Corneal flat mount immunostaining

Rats were anesthetized with a combination of ketamine (Pfizer) and Dexdomitor (Orion Pharma) and euthanized by intracardial injection of pentobarbital. The cornea was dissected for whole-mount immunofluorescent staining and then fixed in cold acetone (− 20 °C) for 30 min. The fixed samples were washed three times in PBS for 30 min each and blocked for 2 h with 10% normal goat serum at room temperature. Primary antibodies against CD31 (1:300, ab24590; Abcam) and collagen IV (Coll IV) (1:300, ab19808; Abcam) were added and incubated overnight at 4 °C. Following PBS washing three times for 30 min, the each, specimens were incubated with secondary antibody (1:1000, Alexa Flour 488-Abcam) and (1:1000, DLlight 549-Abcam) overnight at 4 °C. The samples were then washed in PBS for 1 h with gentle agitation, ProLong Diamond antifade reagent (Thermo Fisher Scientific).

## Results

### L-VGCC protein in silico modeling and binding site evaluation

**Fig. 1:**
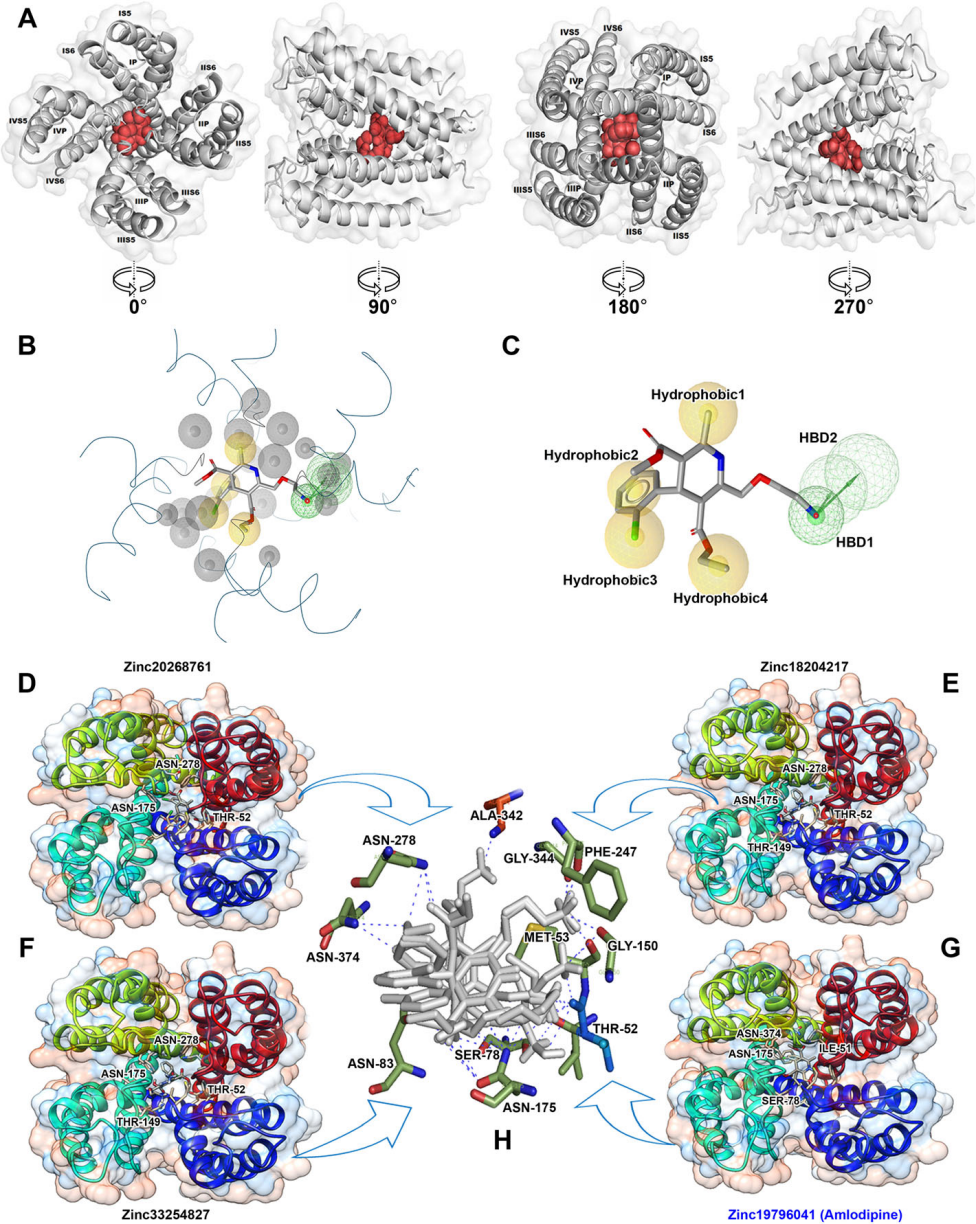
In silico analysis of L-VGCC active site and pharmacophore features. The L-VGCC protein model of the active site (red) and their key amino acids from different angles 0°, 90°, 180°, and 270° (**a**). The pharmacophore model incorporated six features: two hydrogen bond donors (green color) and four hydrophobic groups (yellow color) (**b**). The location of hydrophobic regions and hydrogen bond donors (**c**). The Zinc20268761 (**d**), Zinc18204217 (**e**), Zinc33254827 (**f**), and reference Zinc19796041 (Amlodipine) (**g**), compounds binding with L-VGCC active site (**h**) residues

In this study, we focused on searching for new compounds specific for the L-VGCC, a model protein for which we previously established [10]. The active site of the L-VGCC model protein was predicted using the CASTp server, which identified the pockets, pocket mouth openings, and cavities in the L-VGCC model protein: pocket id-1, area-333.981, and volume-172.598, respectively. The CASTp server predicted that the binding site of the L-VGCC model protein contained amino acids including Ile-51, Thr-52, Met-53, Ser-78, Phe-79, Leu-82, Asn-83, and Leu-86 in chain-A; Leu-148, Thr-149, Gly-150, Leu-170, Phe-171, Gly-174, Asn-175, Leu-178, Leu-179, and Phe-182 in chain-B; Thr-246, Phe-247, Phe-274, Phe-275, Asn-278, and Ile-279 in chain-C; and Ala-342, Thr-343, Gly-345, Ala-369, Phe-370, Ile-373, Asn-374, and Val-377 in chain-D, all of which exhibit binding interactions with dihydropyridine (DHP) derivatives. The target protein of the active sites and their key amino acids are shown in Fig. 1a.

### Structure-based pharmacophore design

We docked amlodipine to an active site of the L-VGCC model and used the protein-ligand complex in the pharmacophore design. The complex was uploaded into the LigandScout software (Ligand, Vienna, Austria). The pharmacophore model was generated by the LigandScout, which incorporated six features, as shown in Fig. 1b: two HBD (hydrogen bond donors) (green color) and four hydrophobic groups (yellow color). Numerous volumes (gray color) in the model were excluded. The two HBD points were the amino acid group and the hydrogen atoms of the ligand in the directions of SER78 and ILE51, respectively. The four hydrophobic groups were positioned on the benzene ring (Hydrophobic2), the chlorine atom in the benzene ring (Hydrophobic3), one hydrophobic region (Hydrophobic4) located in the carboxyethyl group of the ligand, and other hydrophobic regions (Hydrophobic1) located in the amide benzene ring (Fig. 1c). This pharmacophore model was then confirmed by screening the test database. In the test database, we maintained the compound amlodipine in a complex structure. First, the amlodipine was extorted in addition to hydrogen atoms that were added, and the energy was minimized using the LigandScout. The minimized structure of amlodipine was then added to the test database.

The finalized pharmacophore model was used to screen the Zinc database (http://zinc.docking.org/) for commercially available compounds. The local database compounds (7875) were loaded into the LigandScout tool, and the MMFF94x force field minimized the 3D structures of all compounds. The pharmacophore model then filtered the conformers of every compound. To be measured as a hit, the compound had to fit all the features of the pharmacophore. In the pharmacophore-based virtual screening, 18 hits were identified to be mapped on the developed pharmacophore model (i.e., having pharmacophore features). These initially identified hits were selected for further investigation in docking studies.

### Protein-ligand interactions

The screened pharmacophore hits were used in the molecular docking simulation to model L-VGCC protein using the automated docking program AutoDock Vina in the PyRx virtual screening tool. The docking simulation results showed that the AMLD and hits were docked into dihydropyridine ring fitting in the left. The docked compounds showed better binding energies than AMLD (Table 1). The Zinc67664832 compounds bound with − 8.5 kcal/mol binding energy to ASN-278, ASN-175, THR-52, SER-78, and ILE-51 active site residues. The Zinc20268761 compounds bound with − 8.1 kcal/mol binding energy to THR-52, ASN-175, and ASN-278 active site residues (Fig. 1d). The Zinc18204217 compounds bound with − 7.5 kcal/mol binding energy to THR-52, THR-149, ASN-175, and ASN-278 active site residues (Fig. 1e). The Zinc38735350 compounds bound with − 7.2 kcal/mol binding energy to THR-52, MET-53, GLY-150, ASN-175, PHE-247, and GLY-344 active site residues. The Zinc33254827 compounds bound with − 7.0 kcal/mol binding energy to THR-52, MET-53, SER-78, ASN-83, GLY-150, ASN-175, ASN-278, ALN-342, and ASN-374 active site residues (Fig. 1f). The Zinc19796041 (AMLD) bound with − 6.8 kcal/mol binding energy to SER-78, ILE-51, ASN-175, and ASN-374 active site residues (Fig. 1g).

**Table 1:** List of best compounds and their respective binding energies, surrounding residues, number of H-bonds, and number of amino acid interactions

ZINC ID	SMILES	Binding energies (ΔG kcal/mol)	Surrounding residues	No. of H-bonds	No. of amino acid interactions
Zinc67664832	CCOC(=O)C1=C(NC(=C([C@H]1c2ccccc2Cl)C(=O)OC)C)COCC[N-][NH2+][NH-]	− 8.5	ASN-278, ASN-175, THR-52, SER-78, ILE-51	07	05
Zinc20267861	CCOC(=O)C1=C(NC(=C([C@H]1c2ccccc2Cl)C(=O)OC)C)COCCNC(=O)CCl	− 8.1	THR-52, ASN-175, ASN-278	06	03
Zinc18204217	c1cc(c(cc1Cl)Cl)[C@H](/C(=N\NC(=O)c2ccncc2)/c3c(nc4cc(ccc4n3)Cl)[O-])O	− 7.5	THR-52, THR-149, ASN-175, ASN-278	06	03
Zinc38735350	CCOC(=O)C1=C(NC(=C([C@@H]1c2ccccc2Cl)C(=O)OC)COCC[NH3+])COCC[NH3+]	− 7.2	THR-52, MET-53, GLY-150, ASN-175, PHE-247, GLY-344	07	06
Zinc33254827	Cc1cc2c(cc1C)n(c(=O)c(n2)C)CC(=O)NCc3ccc(cc3OC)OC	− 7.0	THR-52, MET-53, SER-78, ASN-83, GLY-150, ASN175, ASN-278, ALN-342, ASN-374,	12	08
Amlodipine (Query)	CCOC(=O)C1=C(NC(=C([C@@H]1c2ccccc2Cl)C(=O)OC)C)COCC[NH3+]	− 6.4	SER-78, ILE-51, ASN-175, ASN-374	04	04

The results of the L-VGCC and ligand interactions showed that ILE-51, THR-52, SER-78, ASN-83, MET-53, ASN-278, ASN-175, and ASN-374 amino acid residues might play a crucial role in the blockade of L-VGCC channels (Fig. 1h).

### Analysis of pharmacokinetic profiles and ADMET properties

Drug-likeness is estimated by predicting pharmacokinetic parameters and bioactivity values, which is accomplished according to Lipinski’s rule of five: the furthermost “drug-like” molecules have a molecular weight ≤ 500, log P ≤ 5, number of hydrogen bond donors (nOHNH) ≤ 5, and a number of hydrogen bond acceptors (nON) ≤ 10. Compounds that violate more than one of these rules may present limited bioavailability or other complications. These parameters, combined with the PSA, were evaluated using the Molinspiration software (http://www.molinspiration.com). The Molinspiration results revealed that almost all the compounds satisfied Lipinski’s rule of five, but Zinc18204217 had 501.97 MW and one violation. The compound Zinc38735350 had seven hydrogen bond donors and one violation. The overall results suggested compounds (Zinc67664832, Zinc20267861, Zinc18204217, Zinc38735350, and Zinc33254827) satisfied Lipinski’s rule of five. Their relevant drug-likeness properties and bioactivity scores are shown in Table 2.

**Table 2:** Molecular properties of best compounds predicted by Molinspiration tool such as logP, TPAS (topo polar surface area), MW (molecular weight), nON (hydrogen acceptor), nOHNH (hydrogen donor), nviolations, nrotb (rotatable bond), and volume

ZINC ID	Parameters							
	miLogP	TPSA	MW	nON	nOHNH	nviolations	nrotb	Volume
Zinc67664832	− 0.26	128.10	437.90	9	4	0	12	384.13
Zinc20267861	3.49	102.97	485.36	8	2	0	12	414.34
Zinc18204217	− 0.98	123.39	501.97	8	2	1	5	383.76
Zinc38735350	− 0.41	138.39	469.97	9	7	1	14	419.62
Zinc33254827	2.82	82.46	395.46	7	1	0	6	365.40
Amlodipine (Query)	− 0.45	101.51	409.89	7	4	0	10	364.70

Molecular descriptors are an integral part of the pharmacokinetic properties and toxicity of a compound. ADMET properties predicted in silico are used to identify the likelihood that the compounds could be used in human therapeutics [35]. In all the compounds, the ADMET properties were calculated using the admetSAR online server (http://lmmd.ecust.edu.cn/admetsar1/about/). Previously, the blood-brain barrier (BBB), aqueous solubility, human intestinal absorption (HIA), CYP450 inhibition, Caco-2 cell permeability, and AMES toxicity properties were calculated to determine the best compounds [36]. The admetSAR calculations showed that all the compounds were capable of penetrating BBB, HIA, and Caco-2 cell permeability. Moreover, all the compounds, except Zinc18204217 and Zinc33254827, were inhibitors of the P-glycoprotein inhibitor-I and noninhibitors of the P-glycoprotein inhibitor-II, except Zinc33254827. No compound showed any inhibitory effects on the renal organic cation transporter (ROCT), CYP enzymes, and various CYP450 substrates and inhibitors, which play a crucial role in drug metabolism.

Further analyses of drug metabolism showed all the compounds were nonsubstrates of CyP450 2C9 and CYP450 2D6 but substrates of CYP4503A4. The results also showed that most compounds did not inhibit CYP450 enzymes. Importantly, all the compounds showed high CYP-inhibitory promiscuity as inhibitors and noninhibitors of CYP450 enzymes, including 1A2, 2C9, 2D6, 2C19, and 3A4. The compounds were nonsubstrates of two CYP450 substrates, 2C9 and 2D6. The ADMET properties of the five best compounds against L-VGCC are presented in Table S1. Furthermore, no compound showed any acute toxicity or mutagenic effect on the AMES test data and other tests. Based on the availability of the substances, we obtained three of the predicted compounds: Zinc20267861, Zinc18204217, and Zinc33254827.

### In vitro toxicity, efficacy, and dose-response evaluation of the CCBs

As L-VGCC is expressed by microglial cells and is suggested to mediate their proinflammatory activation [8], we selected the well-established mouse brain BV-2 microglial cell line to evaluate the toxicity and efficacy of the newly identified CCBs. The toxicity of the compounds was evaluated in BV-2 cells based on fluorescent live/death staining (Fig. S1), which stains living cells fluorescent blue and dead cells fluorescent green. All three compounds tested, Zinc20267861, Zinc18204217, and Zinc33254827, showed low toxicity in their respective concentrations ranging from 1–10 μM (Fig. S1A-C**)**. Cells treated 10% DMSO in PBS were used as a negative control (Fig. S1D), and Triton-X100 0.05%-treated cells served as a positive control for cell death (Fig. S1E). The number of dead cells per field was counted and averaged, which confirmed a statistically insignificant correlation (*P* > 0.05) between the nontreated cells and the compounds under investigation (Fig. S1F).

**Fig. 2:**
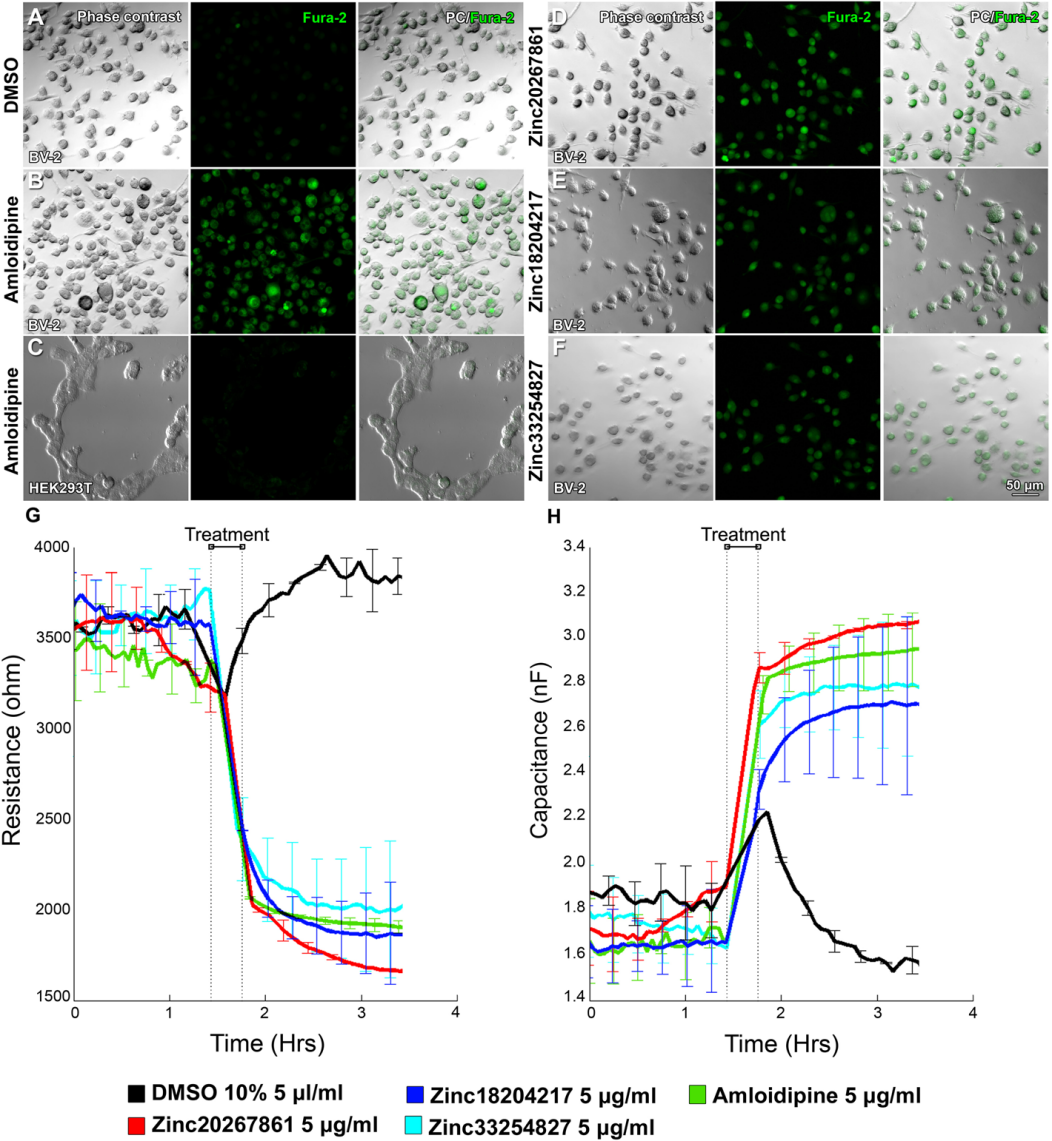
Detection of Ca^2+^ signals and capacitance in BV-2 cells with and without an L-VGCC blockade. Fura-2 Ca^2+^ signals in untreated BV-2 cells used as the negative control (**a**). Amlodipine treated BV-2 cells used as the positive control (**b**). Amlodipine treated HEK293T cells used as assay specificity control (**c**). BV-2 cells treated with Zinc20267861 (**d**), Zinc18204217 (**e**), and Zinc33254827 (**f**). Electric cell-substrate impedance sensing (ECIS) evaluation of resistance (4 kHz) (**g**) and capacitance (16 kHz) (**h**) in BV-2 cells treated with Zinc20267861, Zinc18204217, Zinc33254827, and amlodipine 10 μM; 10% DMSO in PBS 5 μl/ml used as control over the 4-h period (*n* = 4)

We next evaluated the effects of the CCBs on L-VGCC in BV-2 microglial cells using FURA2 staining by observing the presence of a fluorescent signal of a Ca^2+^ free Fura-2 signal at 380 nm excitation (Fig. 2a–f).

The DMSO-treated BV-2 cells used as a control and demonstrated minimal fluorescence due to FURA2 saturation with Ca^2+^ and an excitation shift to 340 nm (Fig. 2a). Strong fluorescence of Ca^2+^ free FURA2 was detected in amlodipine-treated BV-2 cells (Fig. 2b) that were used as a positive control. The assay specificity control utilized HEK293FT cells, which lack L-VGCC and when treated with amlodipine did not respond to the L-VGCC blockade, as observed by the maintained Ca^2+^ saturation of the FURA2 dye, producing noise level signals in HEK293FT cells (Fig. 2c). Zinc20267861 (2.0 μM) (Fig. 2d), Zinc18204217 (2.0 μM) (Fig. 2e), and Zinc33254827 (2.5 μM) (Fig. 2f) produced strong fluorescence at 380 nm excitation, which indicated the presence of Ca^2+^ free FURA2 that was comparable in strength to the amlodipine-treated group. These results indicated the presence of L-VGCC in BV-2 cells, and a decrease in intracellular Ca2+ to levels comparable to the amlodipine-treated positive control (Fig. 2b) in response to Zinc20267861, Zinc18204217, and Zinc33254827 treatment (Fig. 2d–f).

We further examined whether CCBs could affect the interactions of BV-2 cells and extracellular matrix, which affects cell motility as well as a changes in capacitance, which were expected due to decrease in intracellular Ca^2+^ and thus overall cell polarity when compared to the culture medium media. We utilized the electrical cell-impedance sensing (ECIS) system, as Lee et al. reported the effective use of ECIS to detect the BV-2 cell migration activity by reading resistance as well as capable of resolving the capacitance [37]. Due to the low sensitivity of the ECIS device, we used higher concentrations of the respective substances (10 μM) in the short-term experiment of 4 h. The ECIS results demonstrated a rapid decrease in the resistance at 4 kHz (Fig. 2g) and increase in the capacitance at 6.4 kHz (Fig. 2h) in BV-2 confluent cultures treated with Zinc20267861, Zinc18204217, Zinc33254827, and amlodipine. These results suggested an effective decrease in intracellular Ca^2+^ due to an observed shift in capacitance, as well as the importance of Ca^2+^ activity for microglial cell adhesion and migration indicated by rapid change in resistance.

**Fig. 3:**
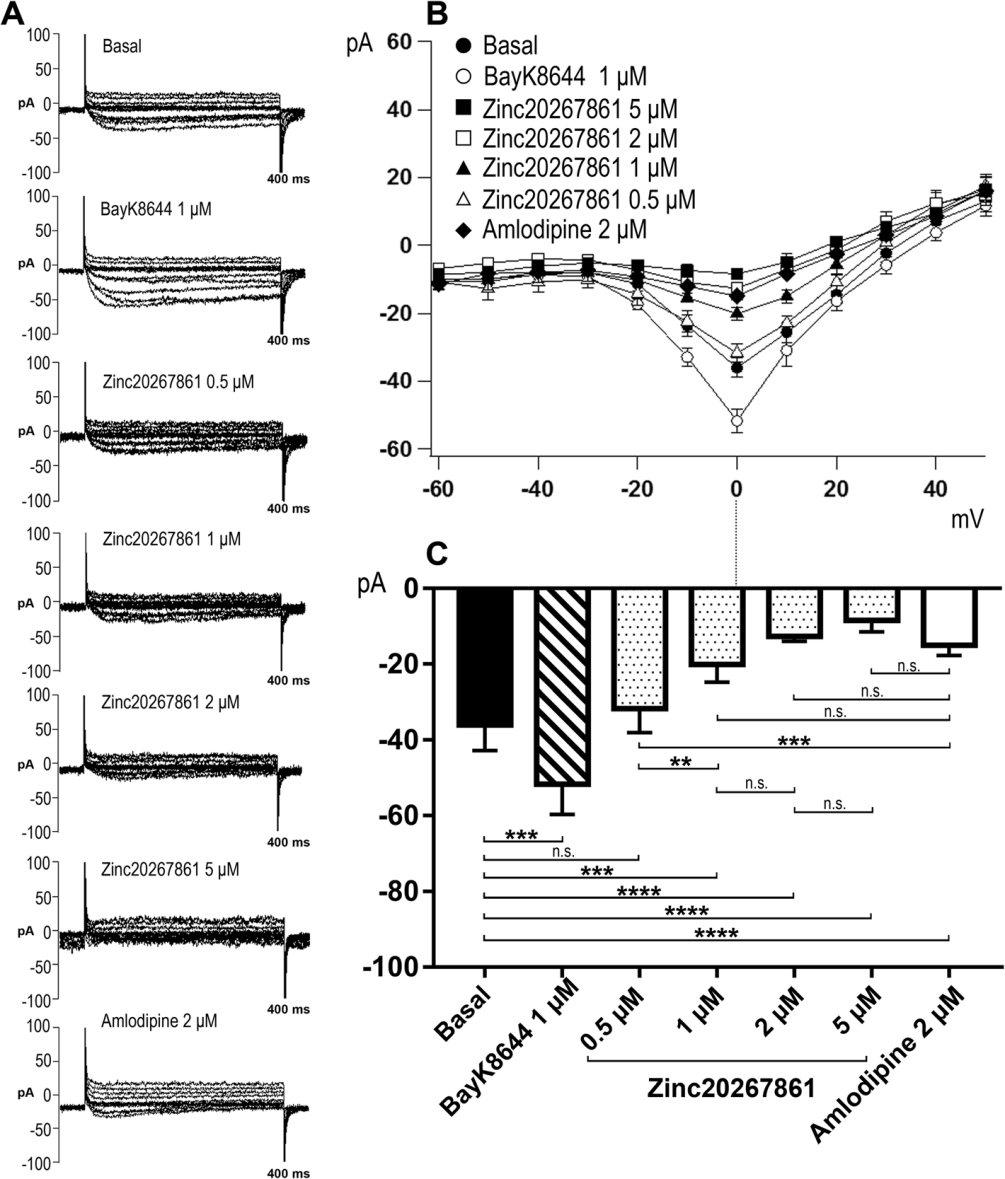
Patch-clamp evaluation on the dose curve of Zinc20267861. Dose curve of 0.5 μM–5 mM of Zinc20267861 with BayK8644 2 μM and Amlodipine 2 μM used as control in smooth muscle cells (SMCs). Representative patch-clamp records (400 ms) of Ca^2+^ current I–V curves (**a**). Ca^2+^ current amplitude picoampere (pA) vs. stimuli voltage (mV) with the channel peak opening is at 0 mV (**b**). Quantitative analysis of pA at channel peak opening of 0 mV (Basal, BayK8644 1 μM *n* = 8; Zinc20268761 2 μM, Amlodipine 2 μM *n* = 6) (**c**). One-way ANOVA test with Tukey multiple comparisons was used to determine statistical significance. n.s. *p* > 0.05; *p < 0.05; **p < 0.01; ***p < 0.001

In addition, we evaluated the efficacy and dose-response characteristics of Zinc20267861 using the voltage patch-clamp method in freshly isolated mouse SMCs. Representative traces of patch-clamp recordings of L-VGCC current evoked by depolarizing voltage steps are presented in Fig. 3a. The I–V curve of Ca^2+^ current is presented in Fig. 3b, Ca^2+^ current amplitude in picoampere (pA) vs. stimuli voltage (mV). Channel peak opening was observed at approximately 0 mV (Fig. 3c). Compared with the baseline, SMCs responded to the known L-VGCC agonist BayK8644 (1 μM), producing a significant increase (*P* < 0.001) in the peak L-VGCC current (approximately − 60 pA at 0 mV). We evaluated the dose curve of Zinc20267861 (the concentration response relationship for Zinc20267861 (0.5–5 μM)) treatments and compared them to 2 μM amlodipine. Zinc20267861 (1–5 μM) treatment of SMCs eliminated the currents (to − 15 pA at 0 mV) when compared to baseline (*P* < 0.01). Marked concentration-dependent inhibition of Ca^2+^ current was observed for Zinc20267861. There was no significant difference in the current density between Zinc20267861 (1, 2, 5 μM) and amlodipine (2 μM) treatment (*P* > 0.05) (1–5 μM; *P* < 0.001) compared with baseline.

### Calcium channel blockade leads to decreased F-actin expression and migration capacity of microglia

**Fig. 4:**
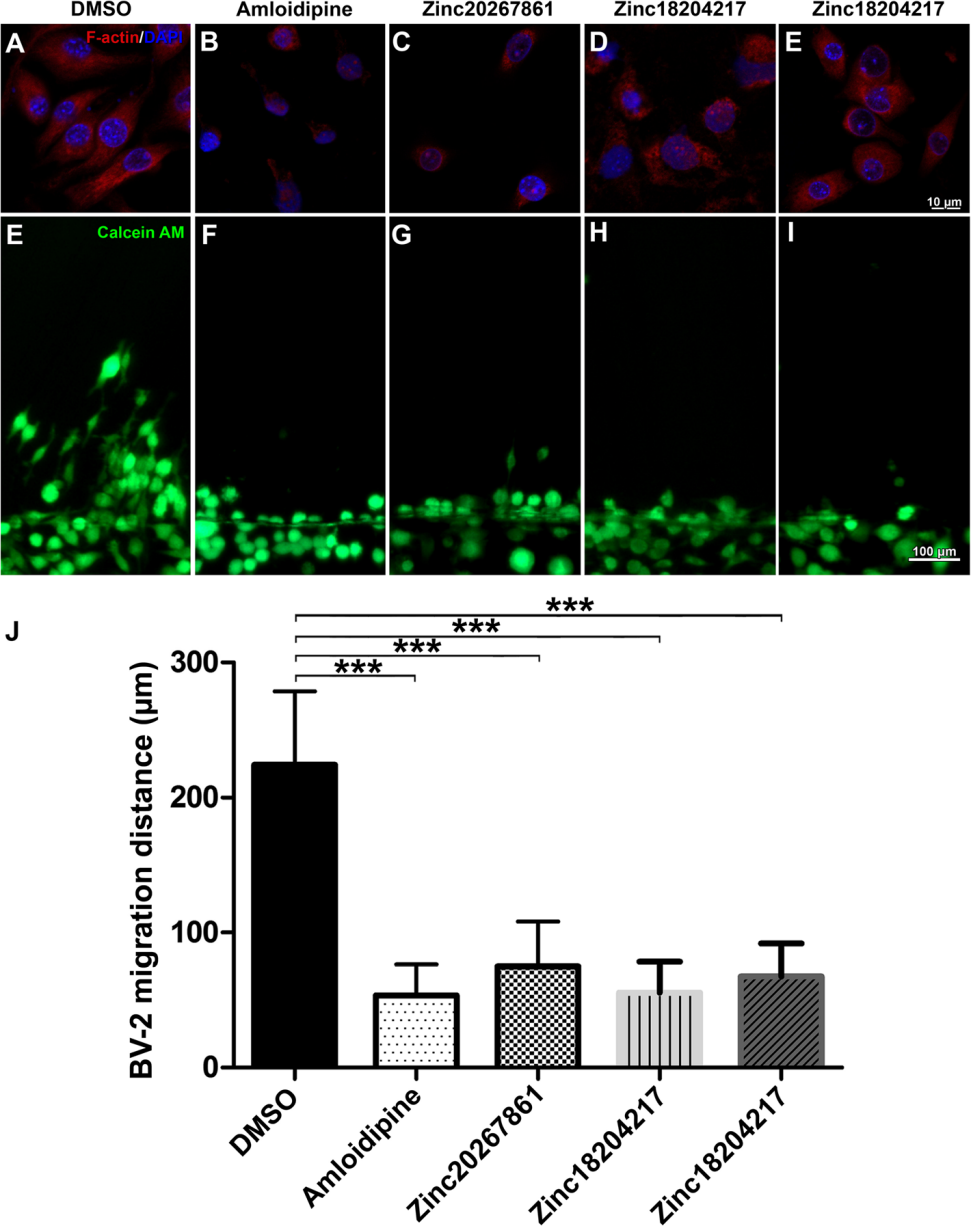
BV-2 cells F-actin expression, structure, and migration capacity affected by L-VGCC blockade. F-actin staining reveal cytoskeleton structure (a–e) and migration capacity in scratch assay as demonstrated by vital Calcein AM staining in BV-2 cells (e–i) with DMSO control treatment (2 μl/ml) (**a**, **e**), Amlodipine (3.5 μM) (**b**, **f**), Zinc20267861 (2.0 μM) (**c**, **g**), Zinc18204217 (2.0 μM) (**d**, **h**), and Zinc33254827 (2.5 μM) (**e**, **i**). Quantification of BV-2 cells migration distance (*n* = 4) (**j**). One-way ANOVA test with Tukey multiple comparisons was used to determine statistical significance. ***p < 0.001

Cytoskeletal organization and migration capacity are critical for the resting surveillance and activating behavior of microglial cells. Increased intracellular Ca^2+^ concentrations have been reported to play important roles in these processes [38, 39]. The ECIS experimental results (Fig. 2 g and h) demonstrating rapid changes in BV-2 cellular resistance and capacitance were also suggestive of a change in cytoskeletal organization. Therefore, we investigated the influence of the L-VGCC blockade on the BV-2 cytoskeleton by visualizing actin-F with phalloidin red (Fig. 4a–e). A disruptive effect on actin-F cytoskeleton formation was evident with amlodipine (3.5 μM) (Fig. 4b), Zinc20267861 (2 μM) (Fig. 4c), Zinc18204217 (2 μM) (Fig. 4d), and Zinc33254827 (2.5 μM) (Fig. 4e). Here, actin-F failed to produce distinct cytoskeletal structures and remained aggregated in the cellular cytoplasm causing a rounded cell morphology. The scratch assay demonstrated prominent cell migration in the DMSO-treated control into the scratch area after 24 h (Fig. 4e), whereas L-VGCC blockade with amlodipine (Fig. 4f) or the candidate substances (Fig. 4g–i) inhibited BV-2 migration capacity. Consistent with the actin-F results (Fig. 4b–e), BV-2 cell L-VGCC blockade (Fig. 4f–i) resulted in a rounded morphology when stained with Calcein AM, whereas DMSO-treated control cells (Fig. 4e) were predominantly more elongated within the outgrowths to the scratch areas. Quantification of migration distance (Fig. 4j) indicated the statistical significance (*P* < 0.001) of the observed effects.

### L-VGCC blockade reduces microglial cell activation capacity when stimulated with LPS or IL-4

**Fig. 5:**
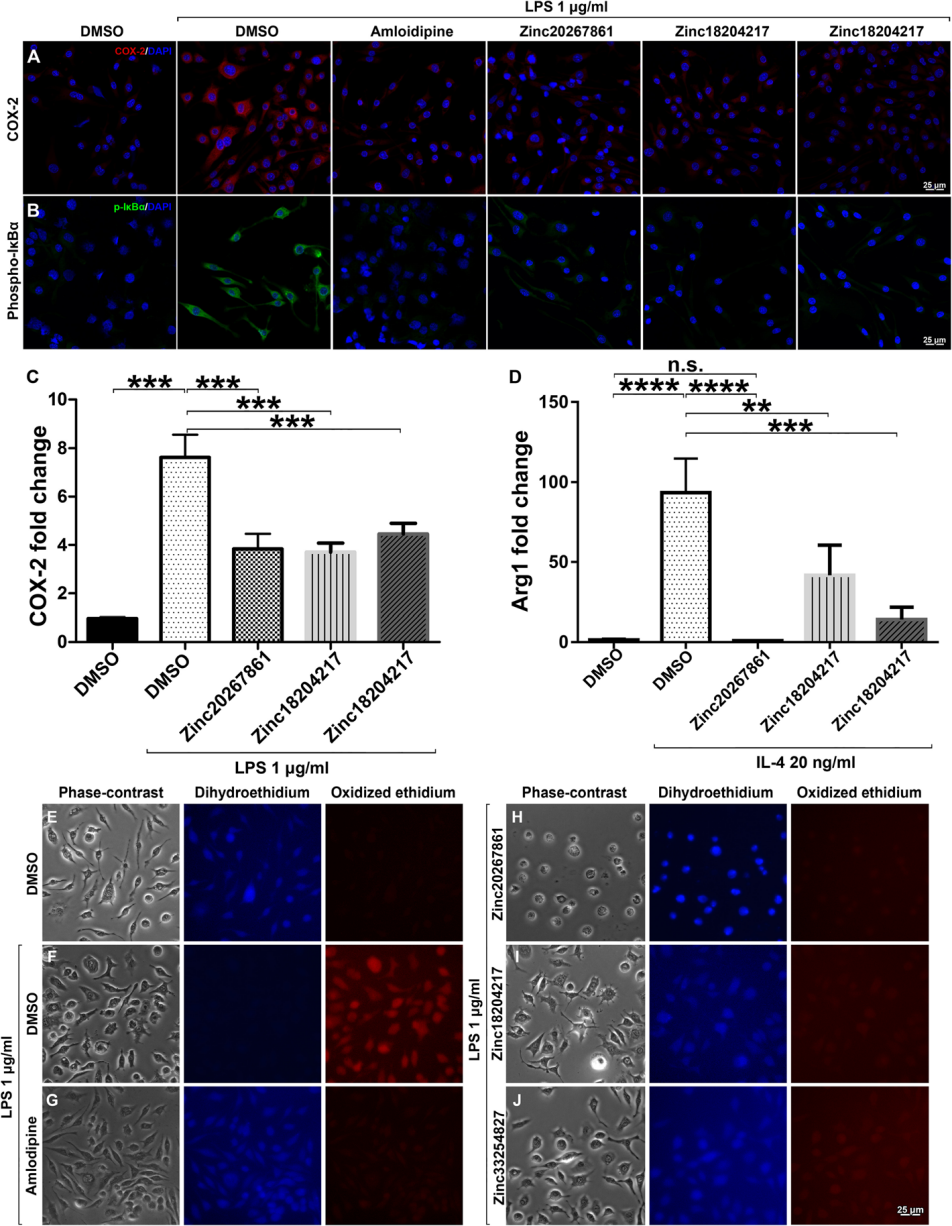
COX-2, phopho-IκBα, and reactive oxygen species expression in LPS-stimulated BV-2 cells with and without L-VGCC blockade. Effects of L-VGCC blockade on COX-2 expression (**a**) and detection of phosphorylated IκBα (**b**) in BV-2 cells stimulated with LPS. Quantitative RT-PCR analysis of COX-2 expression in BV-2 cells treated with DMSO control (2 μl/ml), Zinc20267861 (2.0 μM), Zinc18204217 (2.0 μM), and Zinc33254827 (2.5 μM) stimulated with LPS 1 μg/ml (**c**). Arg-1 expression with the same treatments in BV-2 cells stimulated with IL-4 (20 ng/ml) (**d**) (*n* = 3 with 3 technical replicates). A one-way ANOVA test with Tukey multiple comparisons was used to determine statistical significance. *p* > 0.05; ***p < 0.001. Target genes were normalized to cyclophilin and presented as the fold change of DMSO control. Detection of reactive oxygen species in BV-2 cell culture, minimal oxidation of dihydroethidium in DMSO 2 μl/ml untreated control cells (**e**); strong oxidized ethidium signals detected when stimulated with LPS 1 μg/ml (**e**). Treatment with 3.5 μM of amlodipine (**g**), Zinc20267861 (2.0 μM) (**h**), Zinc18204217 (2.0 μM) (**i**), and Zinc33254827 (2.5 μM) (**j**) demonstrated marked reduction of the oxidized ethidium signals in BV-2 cell culture.

We further examined whether L-VGCC blockade by the new CCBs could prevent microglial cell activation. The treatment of BV-2 cells with Zinc20267861, Zinc18204217, and Zinc33254827, followed by stimulation with 1 μg/ml of LPS, showed reduced expression of COX-2 (Fig. 5a) and phospho-Iκbα (Fig. 5b), which indicated the reduced effect of pro-inflammatory activation comparable to the treatment with amlodipine at 1 μg/ml. RT-PCR analysis of COX-2 expression demonstrated a significant decrease in COX-2 mRNA levels in Zinc20267861 (*P* < 0.001), Zinc18204217, and Zinc33254827 (*P* < 0.001)-treated BV-2 cells with LPS stimulation (Fig. 5c). Interestingly, L-VGCC blockade significantly reduced Arg-1 mRNA levels, when stimulated with IL-4 (20 ng/ml) (Fig. 5d). While the expression of COX-2 and Arg-1 in microglial cells in the context of the M1/M2 activation concept remains controversial [40], these results align with those derived by immunohistochemistry, suggesting that increased Ca^2+^ concentrations are critical for microglial cell activation regardless stimuli.

### Reduced ROS production in BV-2 cell culture with L-VGCC blockade

As calcium signaling can interact with reactive oxygen species (ROS) to promote pro-inflammation in microglia [41], we investigated the effect of L-VGCC blockade on ROS production. ROS production in LPS (1 μg/ml)-stimulated BV-2 cells was evaluated by the conversion of dihydroethidium dye to ethidium by free radicals. The untreated controls demonstrated strong fluorescence in unoxidized dihydroethidium in the 350-nm channel with minimal fluorescence at 595 nm, which indicated the lack of ROS activity (Fig. 5e). In LPS-treated BV-2 cells, strong ROS production was observed, and a predominantly oxidized ethidium signal was detected (Fig. 5f). Treatment with amlodipine (3.5 μM) markedly reduced ROS signaling (Fig. 5g). Treatment with Zinc20267861 (2.0 μM; Fig. 5h), Zinc18204217 (2.0 μM; Fig. 5i), and Zinc33254827 (2.5 μM; Fig. 5j) reduced ROS production similar to the amlodipine treatment. Interestingly, the intensity of the ROS signal was consistent with the predicted binding energy of the substances to L-VGCC; the Zinc33254827 with the least binding energy to L-VGCC produced a stronger ROS signal (Fig. 5j).

### Zinc20267861 subconjunctival treatment reduced the lesion size, fluorescence leakage, inflammatory cell infiltration, neovascularization, and reduced the expression of COX-2 and phospho-IκBα in retinal pigment epithelia of laser CNV mice

**Fig. 6:**
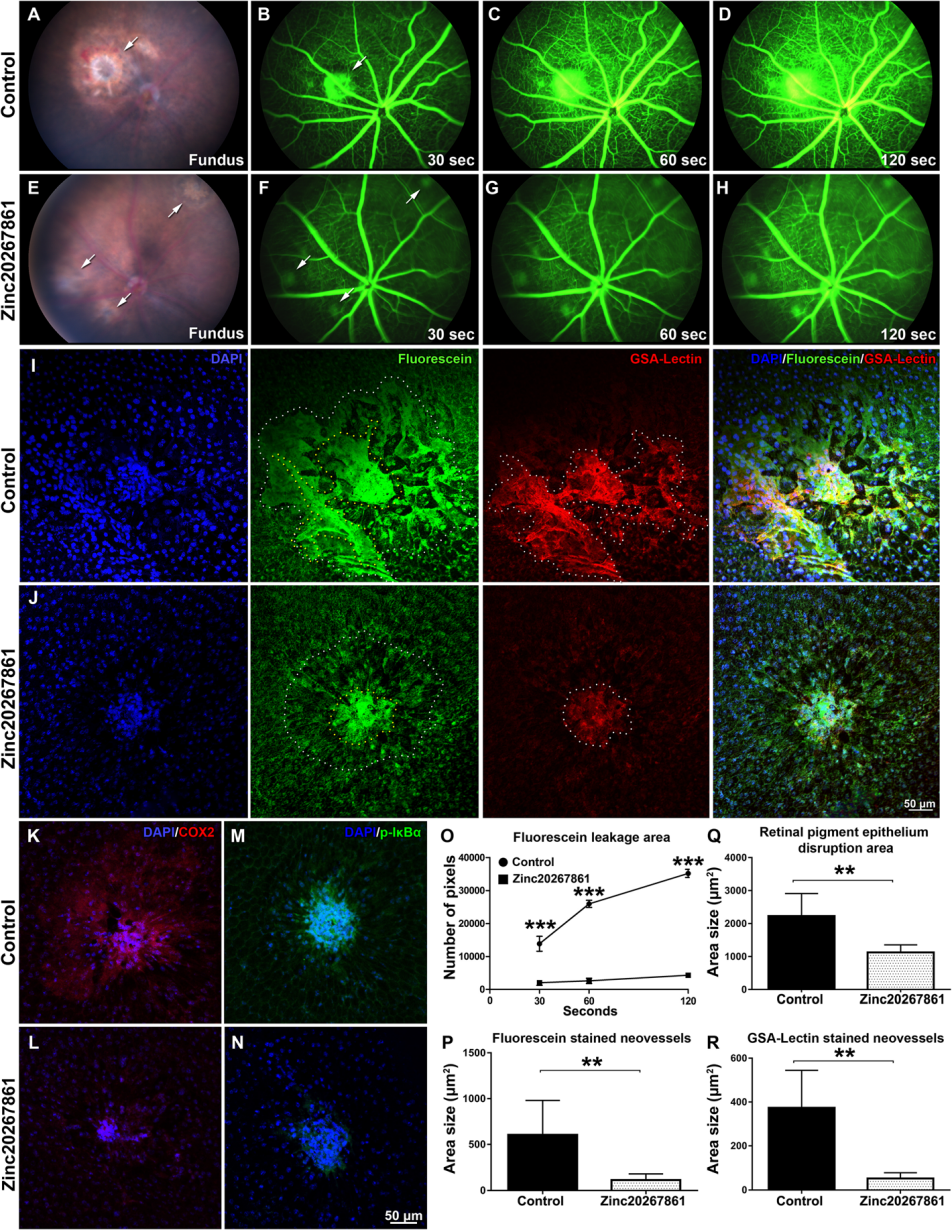
Effects of L-VGCC blockade on laser-induced CNV and expression of COX-2 and phospho-Iκbα in mice. Fundus in vivo images of vehicle-treated laser CNV fundus 5 days after induction of the model (**a**) and Zinc20267861 (10 μg) subconjunctival treated eyes (**e**). Arrows indicate laser burn spots. In vivo fluorescent angiography in vehicle-treated laser CNV animals 30 (**b**), 60 (**c**), and 120 (**d**) after subcutaneous injection of fluorescein sodium 1 mg/kg and same time points (**f–h**) for Zinc20267861 (10 μg) subconjunctival treated eyes. Arrows indicate fluorescein leakage areas. Ex vivo evaluation of the laser spot sizes in retinal pigment epithelia choroid-scleral complexes (RCSC) flat mounts in control (**i**) and Zinc20267861 (10 μg) treated eyes (**j**) counterstained with GSA-lectin. Immunofluorescent detection of COX-2 (**k**, **l**) and phospho-Iκbα (**m**, **n**) in RCSC flat mounts in control and Zinc20267861**-**treated eyes. Quantitative analysis of fluorescein leakage area over time in vivo (**o**). A one-way ANOVA test with Tukey multiple comparisons was used to determine statistical significance. ***p < 0.001; (*n* = 3). Ex vivo quantitative analysis of fluorescein-stained neovessels (*n* = 6) (**p**), retinal pigment epithelia disruption area (*n* = 6) (**q**), and GSA-lectin stained neovessels (*n* = 6) (**r**). Student *t*-test was used to determine statistical significance between two groups. *p < 0.05; **p < 0.01; ***p < 0.001

The in vivo effects of the new CCB (Zinc20267861) were further evaluated using models of laser-induced CNV. CNV was induced by laser injury to the RPE and Bruch’s membrane in mice. The compound Zinc20267861 (10 μg) was delivered into the eye by a single subconjunctival injection, with control mice receiving a single injection of the solvent. The mice were examined 5 days post laser induction. The fundus examination demonstrated reduction of the lesion size in the Zinc20267861-treated eyes compared with the vehicle-treated CNV control (Fig. 6 a and e). Fluorescent angiography demonstrated a marked reduction of fluorescein leakage from laser lesions in the Zinc20267861-treated animals at 30, 60, and 120-s post fluorescein 1 mg/kg injection compared with the vehicle-treated controls (Fig. 6b–d and f–h). Fluorescein and GSA-lectin-stained areas were apparently reduced in the Zinc20267861-treated group compared with the control group in ex vivo observations of RPE/choroid/scleral (RCSC) flat mounts (Fig. 6 i and j). The immunostaining results revealed increased expression of COX-2 and phospho-Iκbα in the laser spots and surrounding area in the vehicle-treated controls (Fig. 6 k and m), which were markedly reduced by Zinc20267861 treatment (Fig. 6 l and n). Quantification showed the size of the leakage area increased over time, indicating a significant decrease in leakage area in the Zinc20267861-treated eyes at 30 (*P* < 0.001), 60 (*P* < 0.001), and 120 (*P* < 0.001) s time points (Fig. 6o). The ex vivo RCSC flat mount fluorescein-stained neovessels, RPE disruption area, and GSA-lectin-stained area were significantly (*P* < 0.01) reduced in the Zinc20267861-treated group compared with the control group (Fig. 6p–r). Therefore, our results indicated that targeting the calcium-NFκB signaling pathway might be an effective solution to treat CNV pro-inflammatory eye disorders.

### L-VGCC blockade suppresses microglial infiltration in laser CNV mice treated with Zinc20267861

**Fig. 7:**
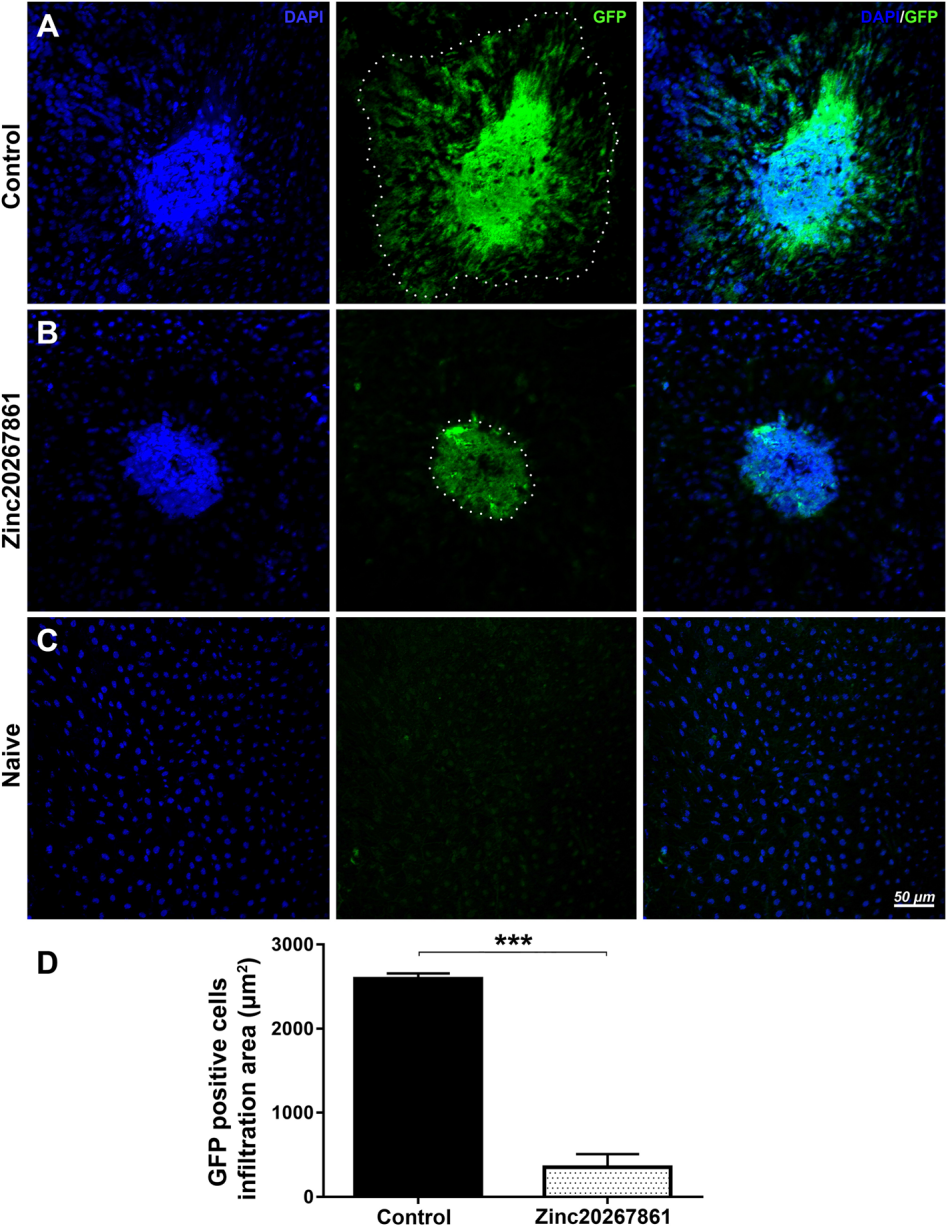
GFP-positive microglia cells and monocytes infiltration of laser CNV spot ex vivo in CX3CR1^gfp/+^ mice with L-VGCC blockade. Retinal pigment epithelia choroid-scleral complexes (RCSC) flat mounts indicate infiltration with GPF-positive cells of the laser CNV spot in vehicle-treated control CX3CR1^gfp/+^ animals (**a**) or Zinc20267861 (10 μg) (**b**) subconjunctivally treated eyes 5 days after model induction. Naive control CX3CR1^gfp/+^ mice RCSC flat mounts shows minimal presence of GFP-positive cells (**c**). Quantitative analysis of GFP-positive cell infiltration area (**d**). Student *t*-test was used to determine statistical significance. ***p < 0.001, (*n* = 3)

The effect of Zinc20267861 on microglial cell migration was observed with GFP-expressing microglia in CX3CR1^gfp/wt^ mice. RPE/choroidal flat mounts of CX3CR1^gfp/wt^ indicated strong infiltration with GFP-positive microglial cells to the laser lesion site and surrounding area in vehicle-treated control animals (Fig. 7a). Treatment with 10 μg of Zinc20267861 markedly reduced the density and area of GPF-positive infiltration (Fig. 7b). A few individual GFP-positive cells were visible in the naïve control RPE (Fig. 7c). Quantification of the size of the infiltrated area revealed significant inhibition of microglial cell infiltration into the laser site (*P* < 0.001) (Fig. 7d).

### Topical application of Zinc20267861 suppresses suture-induced corneal neovascularization in rats

**Fig. 8:**
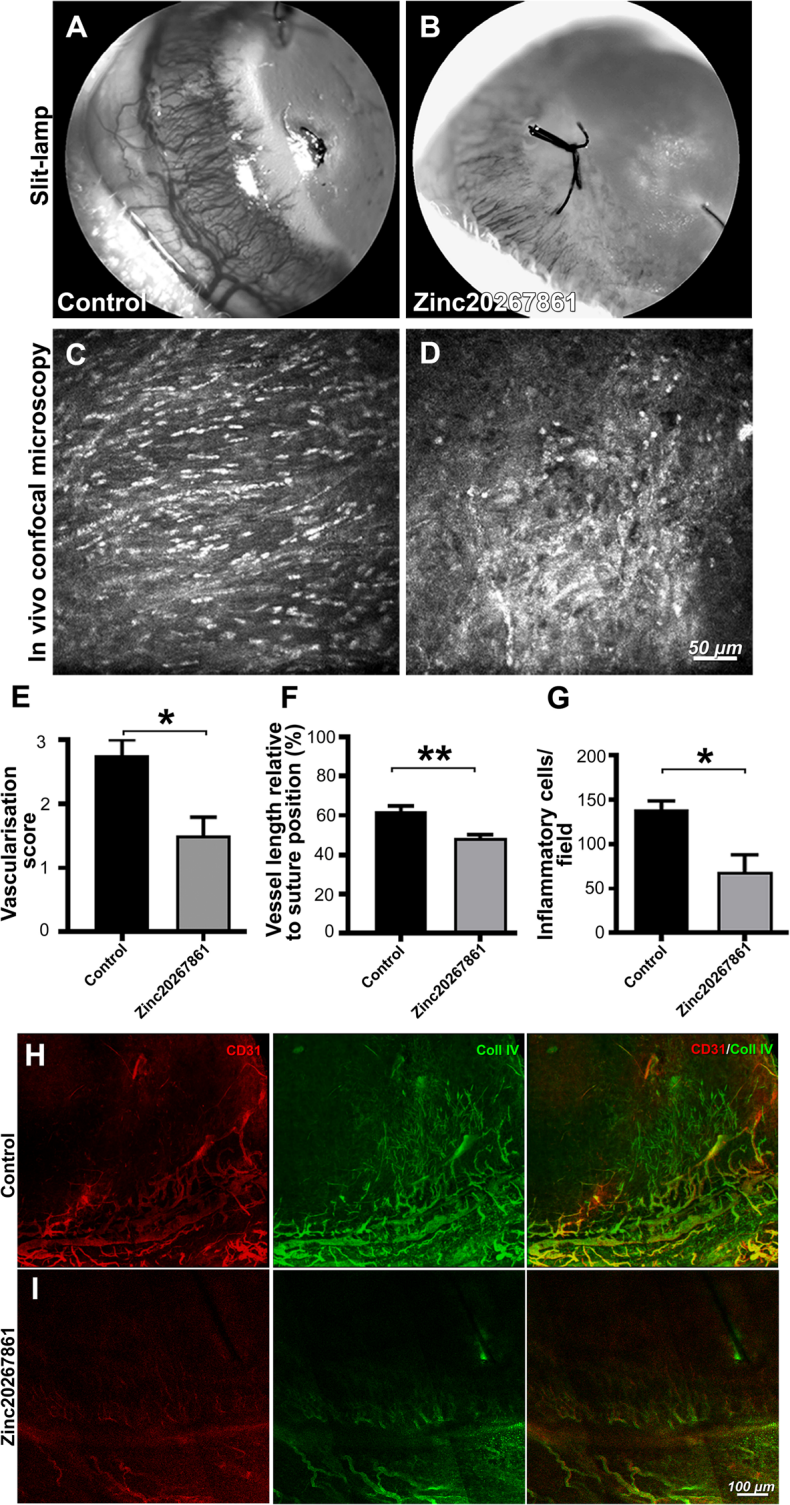
Evaluation of Zinc20267861 topical treatment in rat suture-induced corneal neovascularization model in vivo. Slit-lamp images of the neovascularization of sutured rat corneas at 96-h time point, treated either with vehicle (10% DMSO in 50% PBS/water) (**a**) or Zinc20267861 (5 μg/μl; 10.3 μM in 10% DMSO in 50% PBS/water) (**b**). In vivo confocal microscopy (IVCM) images depicting corneal stromal inflammatory cell infiltration in vehicle-treated controls (**c**) and Zinc20267861 5 μg/μl topical treatment (**d**). Semi-quantitative vascular density and vascular progression score (**e**). Quantification of neovessels length % relative to suture position (**f**). Quantification of infiltrating inflamatory cells per 400 × 400 μm area (**g**). Immunofluorescence staining of CD31 and Coll IV in rat corneal whole mounts 96 h after induction of SI-CNV, treated topically either with vehicle (**h**) or Zinc20267861 (**i**). CD31/Coll IV double-positive signals visualize mature perfused vasculature with developed endothelia. Immature basement membranes are visualized by Coll IV signal only. Student’s *t*-test was used to determine statistical significance in all three quantitative analysis of in vivo data. *p < 0.05; (*n* = 4)

Finally, we investigated the topical effect of Zinc20267861 on suture-induced corneal neovascularization SI-CNV and inflammation in rats. Prominent neovessels had formed in the vehicle-treated eyes (Fig. 8a). Daily topical treatment with 5 μg/ml Zinc20267861 (10.3 μM) markedly reduced the apparent neovasculature, as visualized in the slit lamp images (Fig. 8b). No increased discomfort or complications were observed in rats treated with Zinc20267861 compared with the vehicle treatment. In vivo confocal microscopy (IVCM) evaluation demonstrated reduced inflammatory cell infiltration (Fig. 8c, d). The semiquantitative vascularization score from the slit lamp images was significantly lower in the Zinc20267861-treated corneas (*P* < 0.05) (Fig. 8e). Topical treatment with Zinc20267861 reduced the vascular length by 13% compared with the controls (*P* < 0.01) (Fig. 8f).

Further ex vivo staining with CD31 and collagen IV (Coll IV) revealed numerous (CD31/Coll IV+) neovessels and basement membranes (Coll IV+) in vehicle-treated corneas at 96 h post-suture placement (Fig. 8h). A marked reduction of neovessels by Zinc20267861 topical treatment was observed when compared with the controls (Fig. 8i). Interestingly, fully mature double-positive neovessels as well as Coll IV-positive basement membrane proliferation into the cornea were inhibited by Ca^2+^ blockade. These results clearly indicated the effectiveness of the new compounds for the inhibition of SI-CNV and inflammation and suggested that Ca^2+^-mediated activation is not limited to microglial cells.

## Discussion

Using a structure-based pharmacophore virtual screening approach, we identified novel compounds with properties of third generation L-VGCC blockers that specifically blocked the entry of Ca^2+^ into the cytosol through the α-subunit of L-VGCC. A structure-based pharmacophore was created based on the complex structure of L-VGCC and amlodipine. This pharmacophore model was utilized to screen the ZINC database. The docking and binding energy forecasts were also assessed in the search output. Subsequently, the five compounds that best satisfied all the criteria for the outline of compounds suitable for L-VGCC blockade were identified: Zinc67664832, Zinc20267861, Zinc18204217, Zinc38735350, and Zinc33254827. These compounds had high predicted binding energies, moderate water solubility, and high ADME indexes of drug likeness. Based on the availability of the substances, we tested in vitro three of the compounds, Zinc20267861, Zinc18204217, and Zinc33254827, all of which demonstrated low toxicity and high efficacy in blocking L-VGCC, as well as the capacity to reduce proinflammatory signaling in microglial cells, including ROS production. Previous studies have demonstrated that calcium channel blockers protect against hypertension-related brain damage [42]. Nifedipine has been described to inhibit the expression of inflammatory and fibrogenic molecules in advanced glycation end product-exposed fibroblasts [43]. The L-VGCC blockers nimodipine and verapamil were verified to confer neuroprotective properties and inhibit microglial instigation [44]. Recent studies have shown that nimodipine and verapamil act as neuroprotective agents by mediating anti-neuroinflammatory effects [45]. Cytokines are mediators that are involved in inflammatory, immune, and immunomodulatory functions [46]. Even though inflammatory responses are required for microglial activation, normal neuronal cell functions must be strictly regulated to avoid neurotoxicity.

The elevation of baseline Ca^2+^ is central in the regulation of specific functions in activated microglia [47, 48]. In vitro microglial activation by LPS leads to chronic elevation of Ca^2+^ in microglial cells [49]. Lowering the intracellular calcium with 1,2-bis(o-aminophenoxy)ethane-N,N,N,N-tetraacetic acid (BAPTA) restores much of the signaling efficacy and attenuated the LPS-induced release of NO, TNF-α, IL-6, IL-12, and other chemokines [50, 51]. L-VGCC blockers, such as nimodipine, inhibit NO production and TNF-α, IL-1β, and prostaglandin E2 secretion from LPS-stimulated microglia, were accompanied by reduced degeneration of dopaminergic neurons [52]. Our RT-PCR results in COX-2 and Arg-1 simulated with LPS and IL-4 also indicated that Ca^2+^ influx was required for microglial activation. Furthermore, we presented evidence for the inhibition of NF-κB activation through the blockade of L-VGCC, both in vitro and in vivo. Ca^2+^ influx across Ca^2+^ channels in the plasma membrane led to calcineurin (CN) activation and recruitment of IκB kinases (IKK) to the caspase recruitment domain-containing membrane-associated guanylate kinase protein-1 (CARMA1)-B-cell lymphoma 10 (BCL10) mucosa-associated lymphoid tissue lymphoma translocation protein 1 (MALT1); CARMA1-BCL10- MALT1 (CBM) complex, which in turn triggered the phosphorylation of IκB and release of the active form of NF-κB [53].

The robust results of the experimental data analyses demonstrated reduced microglial cell motility through changes in the expression and structure of actin filaments of the cytoskeleton. Ca^2+^ also affected the remodeling of actin, which is a crucial element of the cytoskeleton and cellular motility [54, 55] that was further confirmed by our ECIS evaluation of BV-2 culture resistance. Although Ca^2+^ does not directly bind to actin, it has been shown to affect the activities of multiple actin regulators, including protein kinase C and calmodulin-dependent kinases [56, 57]. Ca^2+^ signaling also regulates Rho GTPases [58], which is required for the formation of actin bundles for lamellipodia, focal adhesion complexes, and filopodia [59], the major components in cell migration. Moreover, the F-actin severing protein cofilin [60] depends on cytosolic Ca^2+^ for its proper activity. A previous study reported relations between IKK2 activity and actin filaments, in which IKK2 blockade arrested endothelial cell migratory capacity and reduced suture-induced corneal neovascularization [15]. In the current study, the same model was used to test the topical potency and anti-inflammatory effects of Zinc20267861 in rats. These results suggest a complex interplay between Ca^2+^ influx through L-VGCC, NF-κB activation, and actin filament remodeling via BCL10 activity and CBM complex formation [61].

In vivo studies based on a laser CNV model in mice and suture-induced corneal neovascularization model in rats showed that compound Zinc20267861 inhibited neovascularization and inflammation in the retina and choroid in microglia, monocyte, and macrophage activation and migration, supporting its efficacy in reducing the proinflammatory activation in microglia, monocytes, and macrophages. The capacity of topical application in the cornea indicates the potential use of Zinc20267861 in the treatment of corneal inflammatory pathologies.

## Limitations of the study

1. 1,4-Dihydropyridine (DHP) motif (Data S1) used in silico compound screening is specific for L-VGCC [10], but not present in other calcium channel proteins, such as the calcium release-activated channels (CRAC) and transient receptor potential channels (e.g., TRPC3 and TRPV2) [62], thereby the newly identified compounds are expected to target only the alpha subunit of L-VGCC, but not other types of calcium channels. However, we cannot fully exclude the possibility of other interactions.
2. BV-2 cell, while representing many microglial cells properties relevant to this work, such as the expression of L-VGCC and capacity to become activated in response to stimuli, represents a cell line with unavoidable differences from primary brain or retinal microglia cells.
3. We attempted to apply the patch-clamp technique to the microalgal cells (both BV-2 cells and primary brain microglial cells) for current recording of Ca^2+^ activity. However, we could not record the Ca^2+^ currents using our patch clam equipment under baseline conditions or when activated with BayK8644. Therefore, we chose to perform Ca^2+^ channel electrophysiological studies on freshly isolated vascular SMCs. This was done due to the known robust expression of L-VGCC and their responsiveness to the agonist, BayK8644 [63]. Future studies could also examine microglial cells.
4. CX3CR1 is present in both microglia and other inflammatory cell types such as monocytes. Thus, as shown in Fig. 5, we cannot definitively claim whether microglial cells, or other CX3CR1-positive cells, may migrate through a laser burn area with a disrupted Bruch’s membrane [64]. We must note, however, that CX3CR1 shows the strongest expression in microglia than other immune cell types [65]. The use of a recently identified TMEM119 microglia marker tag [66] in mice is preferable, but it was not available to the authors.

## Conclusion

Ca^2+^ influx is required for proinflammatory activation of immune cells, including microglia, through activation of NF-κB via downstream signaling. In eye inflammation, tissue damage and neovascularization occur through inflammatory cell migration. Cell motility is dependent on Ca^2+^ signaling through the reorganization of actin filaments in the cells. Therefore, limiting the access of inflammatory cells to lesion sites may be an effective strategy to reduce ocular tissue damage and pathological angiogenesis. L-VGCC blockade is a promising, effective, and safe method for the reduction of ocular inflammation in both the anterior and posterior segments of the eye. Our in silico model identified three new substances with properties of third generation L-VGCC blockers with high specificity, which were verified in both in vitro and in vivo experiments.

## Statistical analysis

All values are expressed as the mean ± standard deviation (SD) for the respective groups. The statistical analyses are performed using the GraphPad Prism software (https://www.graphpad.com/scientific-software/prism/). The Student’s *t*-test is used to compare two groups. A one-way ANOVA with Tukey multiple comparisons was used to compare multiple groups. A *p* value less than 0.05 was considered significant. The following designations of *p* value were used in the figures throughout the manuscript: n.s. = *p* > 0.05, * = *p* < 0.05, ** = *p* < 0.01, and *** = *p* < 0.001.

## Supplementary information

**Additional file 1.**

## Data Availability

All data generated and analyzed during this study are included in this published article and its supplementary information. Raw datasets for in silico, in vitro, and in vivo experiments are available from the corresponding author upon reasonable request.

## Abbreviations

ADMET: Absorption, distribution, metabolism, elimination, and toxicity
AD: Alzheimer’s disease
AMLD: Amlodipine
BCL10: B-cell lymphoma 10
CN: Calcineurin
CNS: Central nervous system
COX-2: Cyclooxygenase-2
DHE: Dihydroethidium
ECIS: Electric cell-substrate impedance sensing
GPCR: G protein-coupled receptor
HIA: Human intestinal absorption
IKK: IκB kinases
IL-1β: Interleukin-1 beta
IL-4: Interleukin-4
i.p.: Intraperitoneally
LPS: Lipopolysaccharide
L-VGCC: L-type voltage-gated calcium channel
MD: Macular degeneration
MG: Microglia
MALT1: Mucosa-associated lymphoid tissue lymphoma translocation protein 1
MS: Multiple sclerosis
NF-κB: Nuclear factor kappa-B cells
NV: Neovascularization
PD: Parkinson disease
PSA: Polar surface area
ROS: Reactive oxygen species
SOCE: Store-operated Ca^2+^ entry
SI-CNV: Suture-induced corneal neovascularization model
TNF-α: Tumor necrosis factor-alpha
