# Supplementary material for: Discovery of novel L-type voltage-gated calcium channel blockers and application for the prevention of inflammation and angiogenesis
Source: J Neuroinflammation. 2020 Apr 25;17:132. doi: 10.1186/s12974-020-01801-9 (PMC7183139; doi:10.1186/s12974-020-01801-9)
Supplement: Supplementary file 1 — Additional file 1. [file 12974_2020_1801_MOESM1_ESM.docx]

**Discovery of novel** **L-type voltage-gated calcium channel blockers and application for the prevention of inflammation and angiogenesis**

**(Supplementary data)**

Madhu Sudhana Saddala^1^☯, Anton Lennikov^1^☯, Anthony Mukwaya^2^, Yan Yang^3^, Michael Hill^3^, Neil Lagali^2^,
Hu Huang^1^*

^1^ University of Missouri-Columbia, Missouri, United States of America

^2^Department of Ophthalmology, Institute for Clinical and Experimental Medicine, Faculty of Health Sciences, Linköping University, Linköping, Sweden.

^3^Dalton Cardiovascular Research Center, University of Missouri-Columbia, Missouri, United States of America

*Corresponding author:

☯ Anton Lennikov and Madhu Sudhana Saddala have contributed equally to this work.

*Corresponding author:

Hu Huang, PhD

Department of Ophthalmology

School of Medicine

University of Missouri-Columbia

1 Hospital Drive, MA102C

Columbia, MO 65212

Phone: 573-882-9899

[huangh1@missouri.edu](mailto:huangh1@missouri.edu)

**Figure S1:** Evaluation of the toxicity by different doses of Zinc20267861, Zinc18204217, Zinc33254827 in BV-2 cell culture.

**
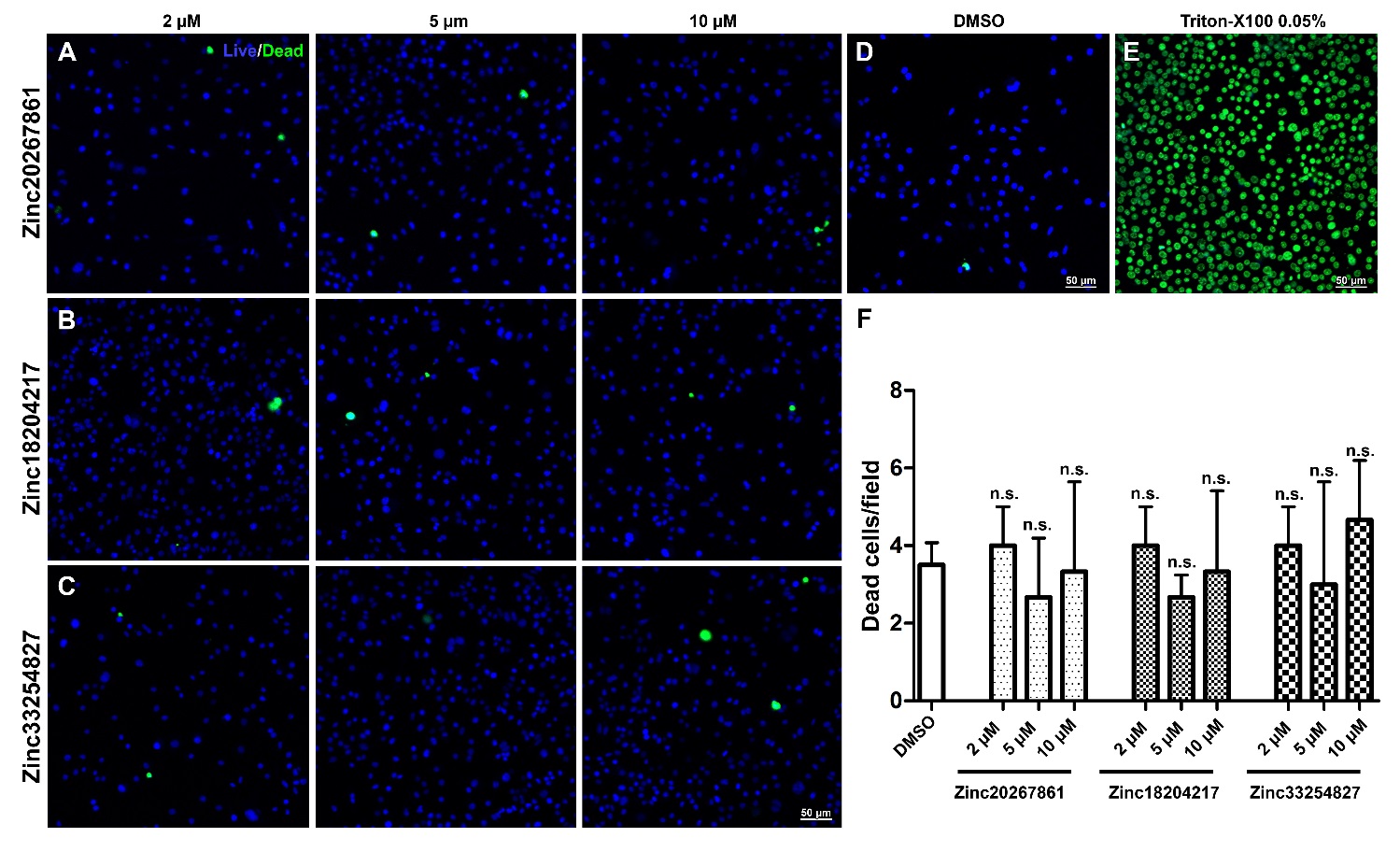
**

Cell death was evaluated by fluorescent live (blue)/dead (green) assay in live culture of BV-2 cells treated with Zinc20267861 (**A**), Zinc18204217 (**B**), Zinc33254827 (**C**) 2, 5, 10 µM. DMSO treated (5 µl) culture used as the negative control (**D**); 30 minutes treatment with Triton-X 0.05% to induce cell death used as assay specificity control (**E**). One-way ANOVA test with Tukey multiple comparisons was used to determine statistical significance. n.s. p > 0.05.

**Data S1.** The 1,4-dihydropyridine (DHP) motif that is specific for L-VGCC.

**CAC1C_Human**

*IS5* IALLVLFVIIYAIIGLELF *290*

*IIS5* LLLLFLFIIIFSLLGMQLF *673*

*IIIS5* VIV**T**TLL**Q**FMFACIGVQLF *1071*

*IVS5* ALLIVMLFFIYAVIGMQVF *1430*

**CAC1C_Human**

*IP* DNFAFAMLTVFQCITMEGWTD *367*

*IIP* DNFPQSLLTVFQILTGEDWNS *710*

*IIIP* DNVLAAMMALFTV**S**T**F**EGWPE *1138*

*IVP* QTFPQAVLLLFRCATGEAWQE *1468*

**CAC1C_Human**

*IS6* WPWIYFVTLIIIGSFFVLNLLVLGVLS *405*

*IIS6* LVCIYFIILFICGNYILLNVFLAIAV *753*

*IIIS6* VEISIFFII**YI**II**I**A**F**F**MM**NIFVGFV *1185*

*IIIS6* EISIFFII**YI**II**I**AFF**MM**NIFVGFVI *1186*

*IVS6* FAVFYFISF**YM**LCAFLI**I**NLFVAVIM *1524*

Residues reported to affect DHPs (motif) antagonist binding and underscored and highlighted in bold. Motif sequence from Saddala et al, Sci Rep. 2017;7:45211.

**Supplementary Tables**

**Table S1:** List of ADMET properties prediction for best compounds by admetSAR tool.

| ADMET Predicted Profile | Zinc67664832 | | Zinc20267861 | | Zinc18204217 | | Zinc38735350 | | Zinc33254827 | | Amlodipine | |
| --- | --- | --- | --- | --- | --- | --- | --- | --- | --- | --- | --- | --- |
| Model | **Result** | **Probability** | **Result** | **Probability** | **Result** | **Probability** | **Result** | **Probability** | **Result** | **Probability** | **Result** | **Probability** |
| Absorption | | | | | | | | | | | | |
| Blood-Brain Barrier | BBB- | 0.8008 | BBB- | 0.6873 | BBB+ | 0.7094 | BBB- | 0.8145 | BBB+ | 0.8197 | BBB- | 0.7987 |
| Human Intestinal Absorption | HIA+ | 0.6291 | HIA+ | 0.9418 | HIA+ | 0.8135 | HIA+ | 0.719 | HIA+ | 0.9557 | HIA+ | 0.5818 |
| Caco-2 Permeability | Caco2- | 0.5875 | Caco2- | 0.5852 | Caco2- | 0.5667 | Caco2- | 0.5423 | Caco2- | 0.5235 | Caco2- | 0.5332 |
| P-glycoprotein Substrate | Substrate | 0.9125 | Substrate | 0.8802 | Non-substrate | 0.6381 | Substrate | 0.9047 | Substrate | 0.7411 | Substrate | 0.91 |
| P-glycoprotein Inhibitor | Inhibitor | 0.7175 | Inhibitor | 0.8963 | Non-inhibitor | 0.9307 | Inhibitor | 0.8103 | Non-inhibitor | 0.514 | Inhibitor | 0.8109 |
|  | Non-inhibitor | 0.9492 | Non-inhibitor | 0.6591 | Non-inhibitor | 0.9063 | Non-inhibitor | 0.8354 | Inhibitor | 0.6806 | Non-inhibitor | 0.7327 |
| Renal Organic Cation Transporter | Non-inhibitor | 0.837 | Non-inhibitor | 0.8318 | Non-inhibitor | 0.8375 | Non-inhibitor | 0.7696 | Non-inhibitor | 0.7806 | Non-inhibitor | 0.812 |
| Distribution | | | | | | | | | | | | |
| Subcellular localization | Mitochondria | 0.8025 | Mitochondria | 0.911 | Mitochondria | 0.7883 | Mitochondria | 0.8836 | Mitochondria | 0.5039 | Mitochondria | 0.8499 |
| Metabolism | | | | | | | | | | | | |
| CYP450 2C9 Substrate | Non-substrate | 0.8751 | Non-substrate | 0.8324 | Non-substrate | 0.8041 | Non-substrate | 0.836 | Non-substrate | 0.8439 | Non-substrate | 0.8224 |
| CYP450 2D6 Substrate | Non-substrate | 0.8361 | Non-substrate | 0.8835 | Non-substrate | 0.8324 | Non-substrate | 0.8812 | Non-substrate | 0.7997 | Non-substrate | 0.8976 |
| CYP450 3A4 Substrate | Substrate | 0.6747 | Substrate | 0.7498 | Substrate | 0.5058 | Substrate | 0.6363 | Substrate | 0.6757 | Substrate | 0.7072 |
| CYP450 1A2 Inhibitor | Non-inhibitor | 0.5511 | Non-inhibitor | 0.5737 | Inhibitor | 0.8817 | Non-inhibitor | 0.5077 | Inhibitor | 0.6506 | Inhibitor | 0.5336 |
| CYP450 2C9 Inhibitor | Non-inhibitor | 0.5763 | Inhibitor | 0.5 | Non-inhibitor | 0.5254 | Non-inhibitor | 0.5248 | Non-inhibitor | 0.6132 | Inhibitor | 0.5397 |
| CYP450 2D6 Inhibitor | Non-inhibitor | 0.822 | Non-inhibitor | 0.8194 | Non-inhibitor | 0.7226 | Non-inhibitor | 0.795 | Non-inhibitor | 0.9677 | Non-inhibitor | 0.77 |
| CYP450 2C19 Inhibitor | Inhibitor | 0.5214 | Inhibitor | 0.6443 | Inhibitor | 0.637 | Inhibitor | 0.595 | Non-inhibitor | 0.5 | Inhibitor | 0.6081 |
| CYP450 3A4 Inhibitor | Inhibitor | 0.6599 | Inhibitor | 0.9233 | Non-inhibitor | 0.7184 | Inhibitor | 0.8054 | Inhibitor | 0.6831 | Inhibitor | 0.8447 |
| CYP Inhibitory Promiscuity | High CYP Inhibitory Promiscuity | 0.6098 | High CYP Inhibitory Promiscuity | 0.6992 | High CYP Inhibitory Promiscuity | 0.6375 | High CYP Inhibitory Promiscuity | 0.7015 | High CYP Inhibitory Promiscuity | 0.5927 | High CYP Inhibitory Promiscuity | 0.6941 |
| Toxicity | | | | | | | | | | | | |
| Human Ether-a-go-go-Related Gene | Strong inhibitor | 0.5074 | Weak inhibitor | 0.8772 | Weak inhibitor | 0.9713 | Weak inhibitor | 0.6203 | Weak inhibitor | 0.9017 | Weak inhibitor | 0.7034 |
| Inhibition | Inhibitor | 0.7273 | Inhibitor | 0.851 | Non-inhibitor | 0.7786 | Inhibitor | 0.7869 | Inhibitor | 0.7004 | Inhibitor | 0.8456 |
| AMES Toxicity | Non AMES toxic | 0.6052 | Non AMES toxic | 0.757 | Non AMES toxic | 0.5798 | Non AMES toxic | 0.7428 | Non AMES toxic | 0.73 | Non AMES toxic | 0.7552 |
| Carcinogens | Non-carcinogens | 0.8107 | Non-carcinogens | 0.86 | Non-carcinogens | 0.7901 | Non-carcinogens | 0.8953 | Non-carcinogens | 0.7918 | Non-carcinogens | 0.865 |
| Fish Toxicity | High FHMT | 0.9787 | High FHMT | 0.9345 | High FHMT | 0.7869 | High FHMT | 0.9908 | Low FHMT | 0.5799 | High FHMT | 0.9628 |
| Tetrahymena Pyriformis Toxicity | High TPT | 0.9965 | High TPT | 0.9965 | High TPT | 0.9658 | High TPT | 0.9972 | High TPT | 0.7988 | High TPT | 0.9951 |
| Honey Bee Toxicity | Low HBT | 0.8085 | Low HBT | 0.8393 | Low HBT | 0.8374 | Low HBT | 0.8202 | Low HBT | 0.7534 | Low HBT | 0.8092 |
| Biodegradation | Not ready biodegradable | 0.9337 | Not ready biodegradable | 0.947 | Not ready biodegradable | 1 | Not ready biodegradable | 0.8686 | Not ready biodegradable | 0.9942 | Not ready biodegradable | 0.9011 |
| Acute Oral Toxicity | III | 0.5896 | III | 0.6151 | III | 0.6055 | III | 0.5847 | III | 0.706 | III | 0.6048 |
| Carcinogenicity (Three-class) | Non-required | 0.5061 | Non-required | 0.6084 | Non-required | 0.6597 | Non-required | 0.6368 | Non-required | 0.6087 | Non-required | 0.5994 |
| Rat Acute Toxicity | 2.6253 | LD50, mol/kg | 2.5301 | LD50, mol/kg | 2.2733 | LD50, mol/kg | 2.6011 | LD50, mol/kg | 2.6354 | LD50, mol/kg | 2.558 | LD50, mol/kg |
| Fish Toxicity | 1.2542 | pLC50, mg/L | 1.2267 | pLC50, mg/L | 1.5712 | pLC50, mg/L | 1.139 | pLC50, mg/L | 1.3713 | pLC50, mg/L | 1.2036 | pLC50, mg/L |
| Tetrahymena Pyriformis Toxicity | 0.6535 | pIGC50, ug/L | 0.6657 | pIGC50, ug/L | 0.7552 | pIGC50, ug/L | 0.8202 | pIGC50, ug/L | 0.3126 | pIGC50, ug/L | 0.7378 | pIGC50, ug/L |
| Solubility | | | | | | | | | | | | |
| Aqueous solubility | -3.5955 | LogS | -3.6784 | LogS | -3.6881 | LogS | -3.7921 | LogS | -3.1128 | LogS | -3.7078 | LogS |
| Caco-2 Permeability | 0.4573 | LogPapp, cm/s | 0.7447 | LogPapp, cm/s | 0.9185 | LogPapp, cm/s | 0.7054 | LogPapp, cm/s | 0.8514 | LogPapp, cm/s | 0.7466 | LogPapp, cm/s |
